# Electrospun Polymer Nanofibers for Biomedical Interfaces: Polymer Selection, Architecture, Bioactivation, and Translational Challenges

**DOI:** 10.3390/polym18212750

**Published:** 2026-11-05

**Authors:** Gianluca Ciarleglio, Maria Gabriella Santonicola

**Affiliations:** Department of Chemical Engineering Materials Environment, Sapienza University of Rome, Via del Castro Laurenziano 7, 00161 Rome, Italy; mariagabriella.santonicola@uniroma1.it

**Keywords:** electrospinning, bioactive nanofibers, polymer functionalization, controlled drug release, surface biofunctionalization, antimicrobial coatings, wound dressing, tissue regeneration, implant/prosthesis coatings, biomaterials

## Abstract

Electrospun nanofibers provide high surface area, interconnected porosity, and extracellular-matrix-like architectures that can be combined with controlled degradation, local therapeutic delivery, and surface biofunctionalization. This review examines bioactive electrospun materials from a materials-design perspective, focusing on the relationships among polymer selection, electrospinning conditions, fiber architecture, bioactive-agent localization, and application-specific performance. Synthetic biodegradable polymers, natural and bio-derived polymers, and hybrid systems are discussed together with random, aligned, multilayer, and core–shell architectures. Bioactivation strategies include direct incorporation of therapeutic agents or functional nanophases, core–shell localization for payload protection and release control, surface functionalization, endogenous-factor recruitment, and stimulus-responsive activation. Three biomedical interfaces are considered as contrasting application models: wound dressings, urological repair, and implant or biomedical-device coatings. Their comparison highlights distinct requirements related to hydration, mechanical loading, microbial exposure, tissue interaction, coating–substrate integrity, and functional lifetime. Particular attention is given to wet-state stability, preservation of bioactive function, sterilization, manufacturing reproducibility, and validation against application-specific failure modes. Overall, the assessment of electrospun biomedical materials should extend beyond as-fabricated morphology and bioactive-agent incorporation to establish whether structural, interfacial, and biological functions are retained under application-relevant conditions.

## 1. Introduction

Bioactive polymeric materials are increasingly designed to combine biocompatibility with the ability to modulate interactions with the biological environment, including cell adhesion, immune response, angiogenesis, and tissue remodeling [1,2,3]. In this context, electrospinning enables the fabrication of polymeric micro- and nanofibers with a high surface-area-to-volume ratio, interconnected porosity, and extracellular-matrix-like morphology. These structural features are particularly relevant for biomedical applications because they influence wetting, protein adsorption, cell–material interactions, mass transport, and local release of therapeutic agents [4,5,6].

Electrospinning is applicable to a broad range of synthetic and natural polymer systems. Synthetic biodegradable polymers, such as poly(ε-caprolactone) (PCL), poly(lactic acid) (PLA), poly-L-lactic acid (PLLA), and poly(lactic-co-glycolic acid) (PLGA), are widely used because of their processability, mechanical properties, and tunable degradation behavior [7,8]. Natural and bio-derived polymers, including collagen, gelatin, chitosan, hyaluronic acid, alginate, silk fibroin, and cellulose derivatives, can provide hydrophilicity and biologically relevant chemical cues, although their use often requires blending, crosslinking, or stabilization to overcome poor spinnability, rapid dissolution, or weak wet-state mechanical performance [9,10,11].

Bioactive electrospun materials are frequently developed as blends, composites, multilayers, or core–shell structures rather than as single-polymer mats. In such systems, the polymer matrix provides structural integrity and influences degradation behavior, while incorporated bioactive molecules, nanoparticles, ceramic fillers, or surface functional groups may impart additional physicochemical or biological functions. Drugs, peptides, proteins, growth factors, antimicrobial agents, and inorganic nanoparticles can be incorporated into the fibers to enable local delivery or provide specific therapeutic functions. Alternatively, fiber surfaces can be modified through plasma treatment, grafting, layer-by-layer assembly, extracellular matrix-derived coatings, or other functionalization strategies to modulate early biological events at the material–tissue interface [12,13].

Despite the large number of electrospun materials proposed for biomedical use, translation remains limited by several materials-related issues. Many nanofibrous systems are optimized and characterized mainly in the dry state, whereas their intended applications involve exposure to aqueous media containing ions and proteins, often under mechanical loading. Under these conditions, swelling, dissolution, hydrolytic or enzymatic degradation, plasticization, loss of mechanical integrity, and instability of bioactive payloads may compromise performance. This is particularly relevant for wound dressings, mucosal materials, urological scaffolds, and implant/device coatings, where the material must remain functional under physiological conditions over time scales compatible with tissue repair or device service life [14,15,16,17].

Wound dressings and skin-regeneration materials represent one of the most active areas of biomedical electrospinning research. Electrospun mats can combine gas exchange, exudate management, conformability, and ECM-like architecture with antimicrobial, anti-inflammatory, angiogenic, or oxygen-delivering functions. Multifunctional fibrous dressings have been developed to target major limitations of chronic and diabetic wounds, including bacterial infection, impaired vascularization, and local hypoxia [11,18,19]. However, these systems must also be evaluated in terms of wet-state stability, release kinetics, cytocompatibility, and long-term biological response.

Electrospun nanofibers are also gaining interest in urological repair and in prosthesis/device coatings, where the biological and mechanical requirements differ substantially from those of wound dressings. Urological materials must withstand persistent hydration, urine exposure, infection risk, and cyclic deformation while supporting regeneration of layered tissues or nerve structures [20,21,22]. In contrast, coatings for dental, orthopedic, or other biomedical devices must remain adherent to the underlying substrate and may be designed to promote tissue integration, limit bacterial colonization, or improve corrosion resistance in metallic implants [23,24,25].

This review examines bioactive electrospun materials from a materials-design perspective, with particular attention to the relationships among polymer selection, electrospinning conditions, fiber architecture, bioactivation strategy, and performance under application-relevant conditions.

Recent reviews have extensively addressed electrospinning technology, polymer classes, drug delivery, tissue-engineering scaffolds, and specific biomedical applications [26,27,28]. Broader multidisciplinary reviews have also highlighted challenges related to scalability, durability, and long-term material stability across electrospun systems [29]. However, the relationships between material selection, structural organization, bioactive-function retention, and failure under use-relevant conditions are less frequently examined across different biomedical interfaces within the same framework. In particular, the effects of hydration, prolonged physiological exposure, sterilization, storage, and mechanical loading are often discussed within individual applications rather than as common materials-design constraints. The present review therefore focuses on the extent to which electrospun materials retain their structural, interfacial, and biological functions after fabrication and during exposure to conditions relevant to their intended use.

Bioactive electrospun materials are defined here as systems in which polymer composition, surface chemistry, incorporated bioactive agents, or responsive behavior are intentionally engineered to modulate a biological process. Extracellular-matrix-like fibrous morphology alone is not considered sufficient to define bioactivity. The selected applications are considered as contrasting models rather than equivalent evidence domains, given differences in the extent and maturity of their experimental validation.

Data-driven approaches are also briefly considered as emerging tools for materials selection and process optimization [30,31]. Particular attention is given to wet-state stability, reproducibility, sterilization, and long-term functionality as key requirements for clinically relevant electrospun materials.

This review focuses on wound dressings, urological constructs, and implant coatings as representative biomedical interfaces with distinct functional requirements. Wound-contact materials emphasize fluid management and temporary tissue protection; urological constructs require compliant support and restoration of tissue barriers during regeneration; implant coatings require stable attachment to a device surface while maintaining the intended biological function. Considering these interfaces together allows recurring relationships among polymer selection, fiber architecture, bioactivation, and exposure-dependent performance to be examined across different functional lifetimes. Cell sheet engineering is discussed within the architectural and interfacial design framework because it introduces the complementary requirement of controlled cellular organization and harvesting.

### Literature Selection and Search Strategy

This critical narrative review examines peer-reviewed literature on the design and biomedical performance of electrospun polymeric materials. Publications were identified through targeted literature searches and reference checking. The search addressed polymer selection, electrospinning parameters, fiber architecture, bioactivation strategies, drug release and functional performance, focusing on wound dressings, urological tissue engineering and implant coatings.

Studies were selected according to their relevance to the relationships among material composition, processing, fibrous architecture, and biomedical function. Original experimental studies were prioritized for information on formulation, physicochemical properties, mechanical behavior, degradation, and biological outcomes. Review articles provided background and supported the interpretation of underlying mechanisms. Comparative analysis considered testing conditions, quantitative outcomes, and the experimental models used, with particular attention to material stability and limitations affecting translation.

## 2. Electrospinning as a Materials Design Strategy

Electrospinning is an electrohydrodynamic fiber-forming process in which solution properties, polymer chain entanglement, jet dynamics, solvent evaporation, and collector configuration jointly determine fiber formation and the final architecture of the material. Its relevance for biomedical materials derives from the ability to generate continuous micro- and nanoscale fibers from a broad range of polymers, composites, and functional additives [5,6,12].

Electrospinning enables control of fiber and mat morphology through the coupling of material, processing, and environmental variables. Fiber diameter, pore size, mat density, orientation, and thickness are influenced by polymer concentration, molecular weight, viscosity, conductivity, surface tension, solvent volatility, applied voltage, flow rate, working distance, and collector configuration [32,33,34,35]. These structural features, in turn, influence wetting, protein adsorption, cell adhesion, fluid transport, mechanical response, and release kinetics, which are relevant to biomedical applications such as tissue engineering, drug delivery, and wound repair [2,36,37,38].

This process–structure coupling is particularly relevant for bioactive materials. A nanofibrous morphology may resemble the extracellular matrix, but morphology alone does not ensure biological function. Under use-relevant conditions, electrospun materials must retain their structure and activity in hydrated, ionic, and protein-rich media and, depending on the application, under mechanical deformation or microbial challenge. Polymer chemistry, fiber architecture, surface chemistry, and payload distribution should therefore be matched to the intended biological environment.

### 2.1. Process–Structure Relationships

**Figure 1:**
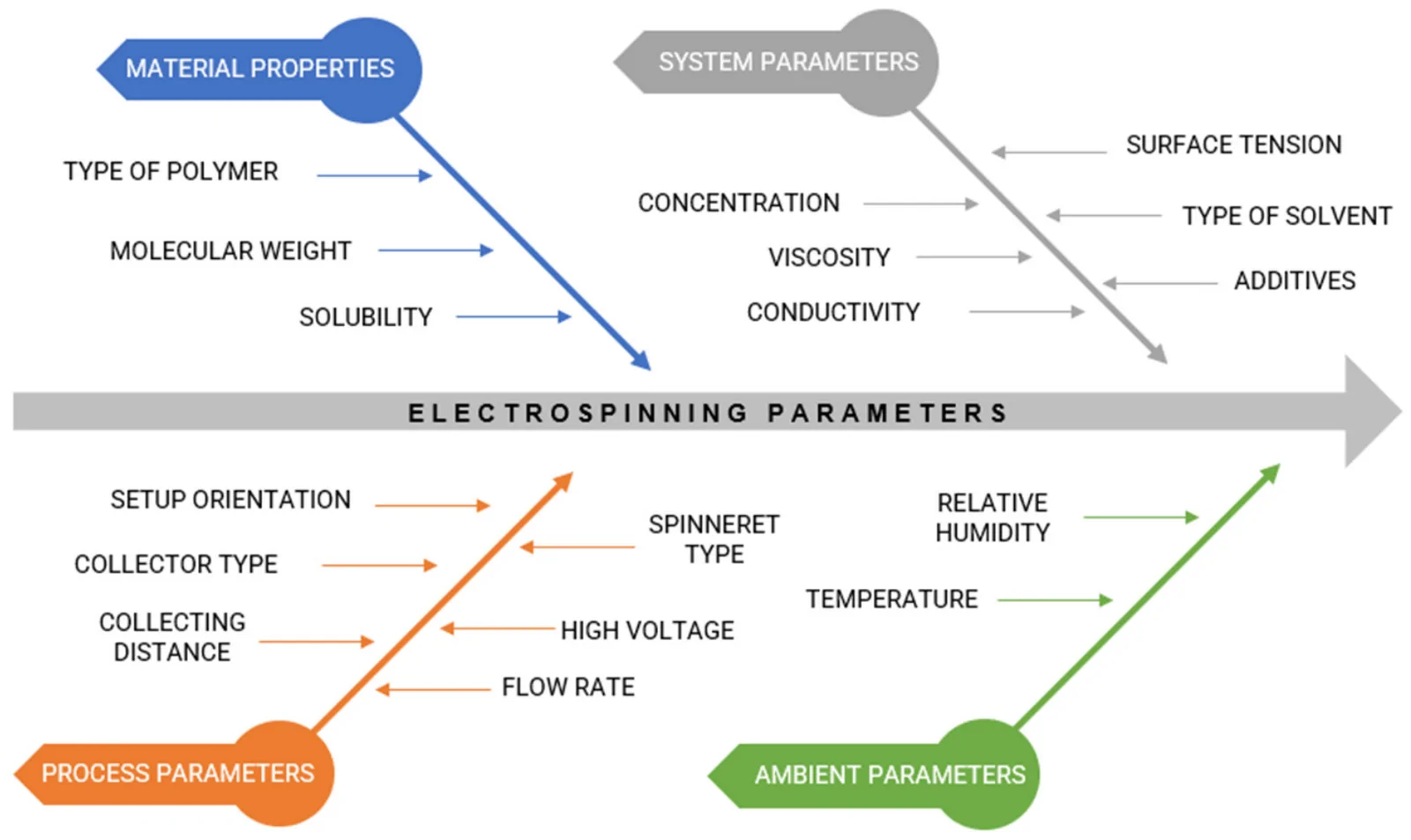
Main material, solution, processing, system, and environmental parameters affecting electrospinning and the resulting fibrous architecture.

Electrospun fiber morphology is governed by material and solution properties, processing parameters, collector configuration, and ambient conditions, which together affect jet formation, stretching, solvent evaporation, solidification, and deposition. Morphology should therefore be considered a process-dependent material property rather than a simple fabrication outcome. The main variables defining the electrospinning window and the resulting fibrous architecture are summarized in Figure 1 [6,39].

Polymer molecular weight, solubility, and polymer–solvent interactions define the range of conditions under which stable fiber formation can occur. Sufficient chain entanglement is required to maintain a continuous charged jet, whereas inadequate entanglement or poor polymer–solvent compatibility can result in bead formation, electrospraying, or heterogeneous deposition. Conversely, excessively high viscosity can limit jet stretching and increase fiber diameter [6,32,33].

Solution properties, including polymer concentration, viscosity, conductivity, surface tension, solvent composition, and additives, further affect electrohydrodynamic jet behavior. Increasing polymer concentration generally promotes chain entanglement and continuous fiber formation until viscosity becomes sufficiently high to limit jet stretching [40,41]. Conductivity affects charge transport and the elongational forces acting on the jet; ionic additives can increase conductivity and promote fiber thinning, although excessive charge density may destabilize the spinning process [42]. Surface tension opposes deformation of the liquid surface and can favor droplet formation when electrostatic and viscoelastic forces are insufficient to sustain the jet [43]. Solvent composition affects polymer dissolution, evaporation rate, phase separation, and fiber solidification. Changes in solvent volatility and polymer–solvent interactions can affect fiber diameter, surface morphology, and defect formation. In addition, the incorporation of a nonsolvent into the spinning solution may induce phase separation, leading to the formation of porous or heterogeneous fiber structures, depending on the polymer–solvent system and evaporation conditions [35,44]. Functional additives can simultaneously modify the electrospinning process and the properties of the resulting fibers. Drugs, peptides, proteins, salts, surfactants, nanoparticles, and ceramic fillers can alter viscosity, conductivity, surface tension, crystallization, and jet stability. Inorganic phases such as ZnO, TiO_2_, hydroxyapatite, bioactive glass, and graphene derivatives can also influence fiber diameter, surface roughness, porosity, and defect formation while introducing specific biological or physicochemical functions [6,45].

Processing parameters translate solution behavior into fibrous architecture. Applied voltage, flow rate, and collecting distance act as coupled variables controlling electrostatic stretching, jet flight time, solvent removal, and deposition. Excessive flow rate or insufficient collecting distance can lead to incomplete solvent evaporation, fused fibers, or morphological heterogeneity, whereas voltages outside the stable processing window may destabilize the Taylor cone or modify jet stretching. Optimization should therefore be performed within a processing window rather than by considering each parameter independently [32,33,34].

Spinneret and collector design provide an additional level of structural control. Single-fluid electrospinning generally produces fibers without intentional radial phase separation, whereas coaxial electrospinning can generate core–shell structures suitable for protecting sensitive payloads and modulating release. Emulsion electrospinning can also generate internally structured fibers depending on formulation and processing conditions. Collector geometry also affects macroscopic organization: static collectors generally produce randomly oriented mats, while rotating drums, mandrels, parallel electrodes, and patterned collectors can promote fiber alignment, tubular geometries, or spatially organized deposition [13,33,46].

Ambient temperature and relative humidity are also critical for process reproducibility. Both parameters affect solvent evaporation, water condensation, phase separation, polymer crystallization, and fiber solidification, and the same formulation can therefore produce different morphologies under different environmental conditions. For biomedical materials, these variations may also affect porosity, wet-state behavior, payload stability, and batch-to-batch consistency. Temperature and relative humidity should consequently be reported and, when possible, controlled during fabrication [47,48,49].

The relevance of the resulting structure depends on the intended biomedical interface.

Randomly oriented fibrous mats can provide surface coverage, conformability, and fluid transport, whereas aligned fibers are particularly relevant when directional cell guidance or anisotropic mechanical behavior is required. Tubular architectures, in contrast, can be designed to reproduce the geometry of tubular tissues and anatomical structures.

Fiber diameter, porosity, mat density, and thickness can further affect wetting, protein adsorption, cell infiltration, permeability, mechanical response, and release kinetics [36,37,45,50]. In conventional densely packed electrospun mats, small pore dimensions can restrict through-thickness cell infiltration and tissue ingrowth, particularly when a three-dimensional regenerative response is required. Strategies including control of fiber packing, sacrificial components, modified collector configurations, post-processing, and three-dimensional expansion have therefore been investigated to increase pore accessibility. However, increased porosity must be balanced against the mechanical integrity, barrier properties, and structural stability required by the intended application [51,52]. Electrospinning should therefore be optimized to obtain fibrous architectures whose morphology, stability, and interfacial properties are compatible with the intended biological environment, rather than simply to produce defect-free fibers.

### 2.2. From Passive Fibrous Mats to Bioactive Systems

Early biomedical applications of electrospun fibers mainly exploited their high surface-to-volume ratio, interconnected porosity, and structural similarity to the extracellular matrix, supporting their use as scaffolds and local drug-delivery materials [12,53]. More recent systems increasingly combine these structural features with specific biological functions, including antimicrobial, anti-inflammatory, antioxidant, pro-angiogenic, hemostatic, or regenerative activity [26,27,54,55].

Bioactive agents or functional components can be introduced into electrospun materials through direct incorporation, core–shell loading, or surface functionalization. Direct incorporation allows drugs, proteins, peptides, growth factors, antioxidants, ions, or nanoparticles to be dispersed within the polymer matrix. This approach is experimentally straightforward but can modify solution viscosity, conductivity, crystallization, fiber morphology, degradation, and mechanical behavior. Surface enrichment or weak retention of the active component may also result in an initial burst release, which can be advantageous for rapid antimicrobial action but undesirable when prolonged or dose-controlled delivery is required [45,54].

Core–shell electrospinning allows structural and bioactive components to be spatially separated within the same fiber. The shell can protect the inner phase and regulate diffusion, whereas the core can contain hydrophilic or labile therapeutic agents [56,57]. Surface functionalization instead localizes the bioactive function at the material–environment interface. Plasma treatment, grafting, layer-by-layer assembly, and immobilization of biologically active molecules can modify early protein-, cell-, or bacteria–material interactions without requiring bulk release [38,58].

The presence of a bioactive agent within an electrospun material does not, however, demonstrate functional bioactivity under application-relevant conditions. The active component must retain its function after processing, sterilization, storage, and hydration, and must remain accessible or be released at a concentration and over a time scale compatible with the intended biological response. At the same time, the fibrous architecture must retain sufficient structural and mechanical stability in the relevant physiological environment [27,54,55].

The main relationships among material formulation, electrospinning conditions, fibrous structure, physiological exposure, and functional performance are summarized in Table 1. These variables are strongly interdependent, and changes introduced at the formulation or processing stage can affect not only fiber morphology but also wet-state stability, payload distribution, release behavior, and biological performance.

As shown in Table 1, the properties of electrospun biomedical materials result from coupled formulation, processing, structural, and environmental variables rather than from individual parameters acting independently. Optimization should therefore consider whether the fibrous architecture and bioactive function are retained after fabrication and subsequent exposure to physiological conditions. Polymer selection represents the first material-level decision within this design framework and is discussed in the following section.

## 3. Polymer Selection for Bioactive Electrospun Nanofibers

Polymer selection is a key factor in the design of bioactive electrospun nanofibers, influencing solution spinnability, fiber formation, mechanical properties, degradation, wet-state stability, and bioactive-agent release. These characteristics depend on polymer chemistry, molecular weight, crystallinity, hydrophilicity, and polymer–solvent interactions [26,28,59]. Polymer selection should therefore reflect the intended biomedical environment, since wound dressings, urological scaffolds, and implant coatings operate under markedly different conditions of hydration, mechanical loading, microbial exposure, and required functional lifetime.

**Figure 2:**
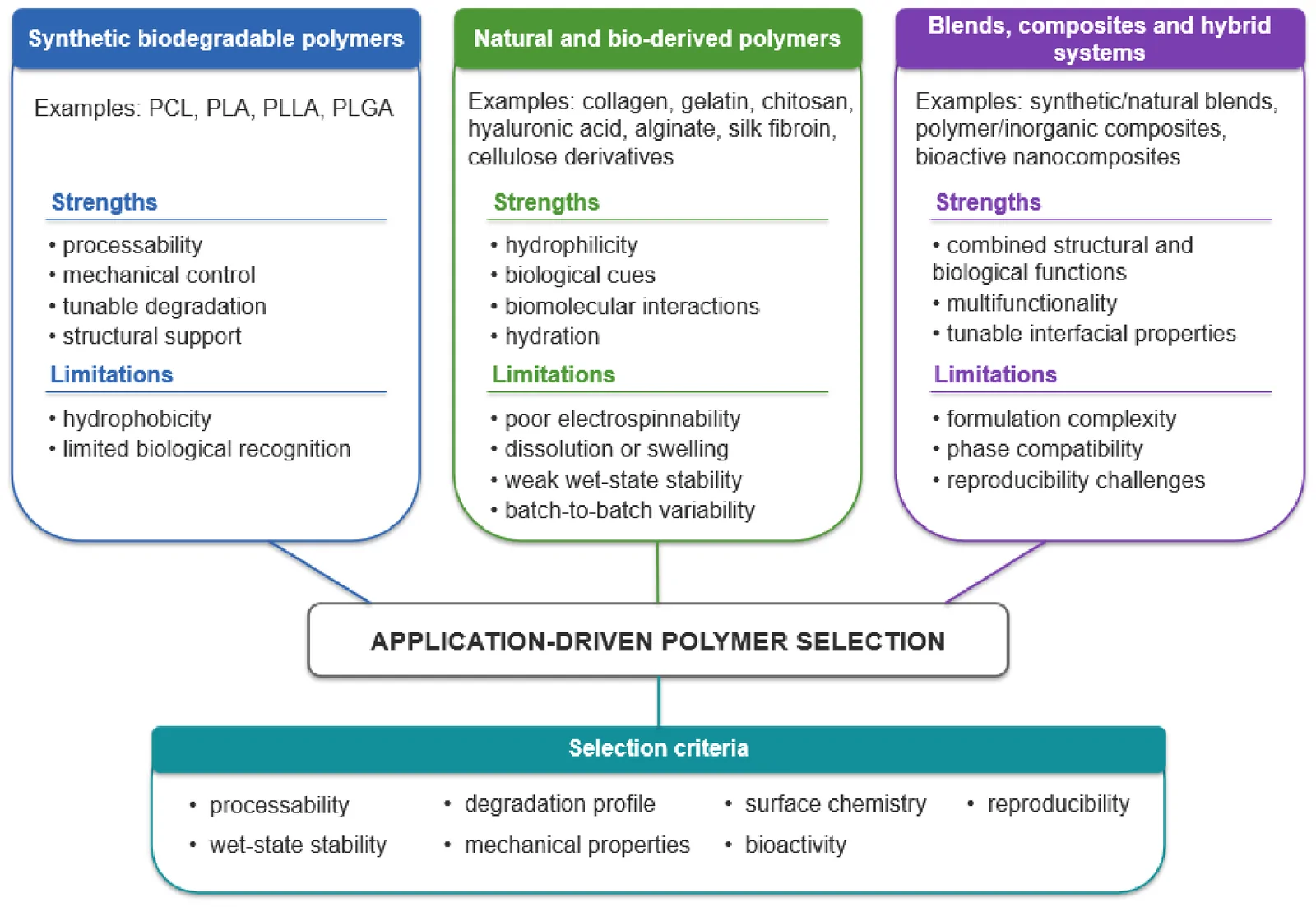
Main polymer classes and materials-selection criteria for bioactive electrospun nanofibers.

From a materials-design perspective, polymers used for bioactive electrospun nanofibers can be broadly classified as synthetic biodegradable polymers, natural or bio-derived polymers, and hybrid systems comprising polymer blends, composites, and combinations with inorganic or bioactive phases. Synthetic polymers generally provide better process control, mechanical stability, and tunable degradation, but often require surface modification, blending, or bioactive loading to improve interactions with the biological environment. Natural and bio-derived polymers provide hydrophilicity and biologically relevant functional groups, but may show limited spinnability, rapid dissolution, poor wet-state mechanical stability, or greater batch-to-batch variability. Combining different polymeric and non-polymeric phases can therefore be used to balance processing, structural stability, degradation, and biological function [28,38,55]. The main polymer classes and selection criteria considered in this review are summarized in Figure 2.

### 3.1. Synthetic Biodegradable Polymers

**Figure 3:**
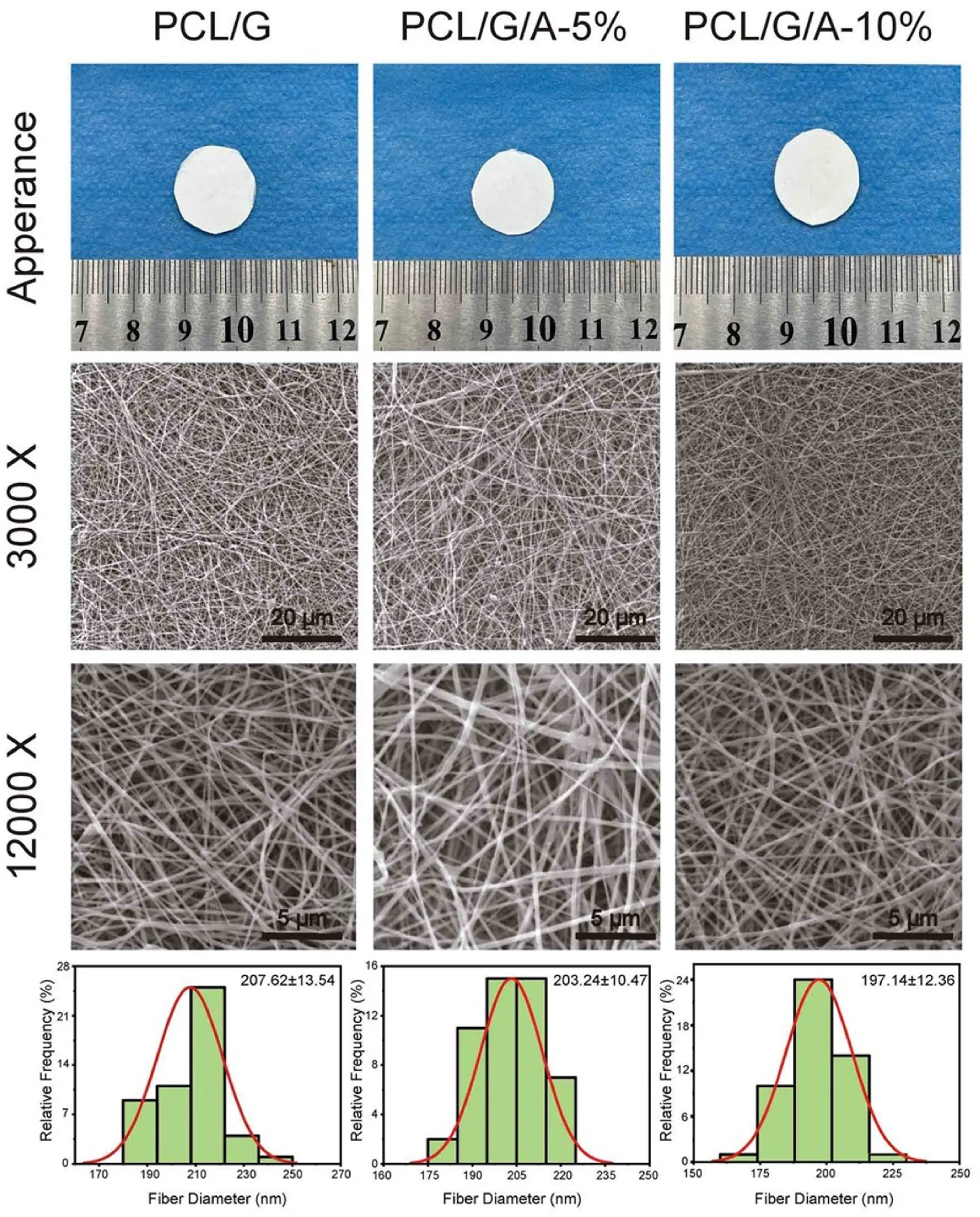
Effect of bioactive loading (arbutin) on the morphology of PCL-based electrospun nanofibers. Representative SEM images and fiber-diameter distributions of PCL/gelatin nanofibers containing different arbutin concentrations. Reprinted from Du et al. [60] under CC BY 4.0.

Biodegradable aliphatic polyesters, including poly(ε-caprolactone) (PCL), poly(lactic acid) (PLA), poly(L-lactic acid) (PLLA), and poly(lactic-co-glycolic acid) (PLGA), are widely used in electrospun biomedical materials due to their processability and tunable mechanical and degradation properties. Their molecular weight, composition, crystallinity, and thermal properties influence fiber formation, hydrolytic degradation, and the functional lifetime of the resulting materials. PCL is characterized by a low glass-transition temperature, semicrystalline structure, high ductility, and relatively slow hydrolytic degradation. These properties favor the fabrication of flexible electrospun mats with long-term structural stability, which is advantageous when mechanical integrity must be maintained during extended physiological exposure. However, its relatively hydrophobic surface can limit wettability and cell–material interactions. PCL is therefore frequently combined with natural polymers, bioactive compounds, or surface modifications to improve interfacial biological performance. For example, electrospun PCL/gelatin membranes containing arbutin showed ROS-scavenging, antibacterial, and wound-healing-related activity, while retaining a continuous fibrous morphology [60]. The effect of arbutin incorporation on fiber morphology is shown in Figure 3.

PLA and PLLA generally provide higher stiffness than PCL and are used when a more rigid biodegradable fibrous matrix is required. Their mechanical behavior and degradation are strongly affected by stereochemistry, molecular weight, crystallinity, thermal history, and electrospinning conditions. These variables can influence not only fiber morphology but also hydrolytic degradation and the temporal evolution of mechanical properties. PLA-based fibers can also incorporate inorganic phases to modify the biological and physicochemical behavior of the material. Electrospun PLA/nano-hydroxyapatite coatings have been directly deposited onto titanium using a green-solvent electrospinning approach, producing continuous fibrous coatings with composition-dependent morphology and degradation behavior [61]. Related PLA/nano-hydroxyapatite fiber coatings have also been investigated for improving corrosion protection of titanium implants [25,62].

PLGA enables modulation of degradation and release because its lactic/glycolic ratio, molecular weight, and end-group chemistry influence water uptake, hydrolysis, polymer erosion, and local acidification [63]. These properties make PLGA particularly useful in electrospun drug-delivery systems in which diffusion and polymer degradation jointly regulate payload release. In wound-dressing applications, PLGA has been used to fabricate core–shell nanofibers co-delivering zinc oxide nanoparticles and vascular endothelial growth factor, combining antibacterial activity with angiogenic stimulation [18]. In these systems, polymer degradation and fiber architecture jointly regulate the spatial and temporal presentation of bioactive components.

Despite their favorable processing and mechanical characteristics, synthetic biodegradable polyesters generally provide limited intrinsic biological recognition. Their hydrophobicity, surface chemistry, degradation products, and changes in crystallinity and mechanical properties during hydrolysis can affect protein adsorption, cell response, and long-term performance in physiological media. They are therefore commonly used as structural matrices whose interfacial and biological properties are modified through blending, incorporation of bioactive phases, or surface functionalization. Across synthetic biodegradable polyesters, direct comparison of biomedical performance remains difficult because polymer chemistry is frequently accompanied by differences in molecular weight, crystallinity, fiber diameter, mat thickness, processing history, surface chemistry, and degradation conditions. Changes in biological response or release behavior should therefore not be attributed to a single material parameter when several structural and physicochemical variables are modified simultaneously.

### 3.2. Natural and Bio-Derived Polymers

Natural and bio-derived polymers provide chemical functionalities and hydration behavior that differ substantially from those of synthetic aliphatic polyesters. Collagen, gelatin, chitosan, hyaluronic acid, alginate, silk fibroin, and cellulose derivatives contain polar and ionizable functional groups and, depending on their molecular structure and biological origin, may also provide cell-interactive motifs. These characteristics can affect wettability, protein adsorption, cell–material interactions, and water uptake. However, high hydrophilicity, strong intermolecular interactions, limited solvent compatibility, and compositional variability can also restrict electrospinnability and reduce structural stability under physiological conditions [9,10,11,64]. Natural origin should therefore not be considered synonymous with bioactivity or superior biomedical performance.

**Figure 4:**
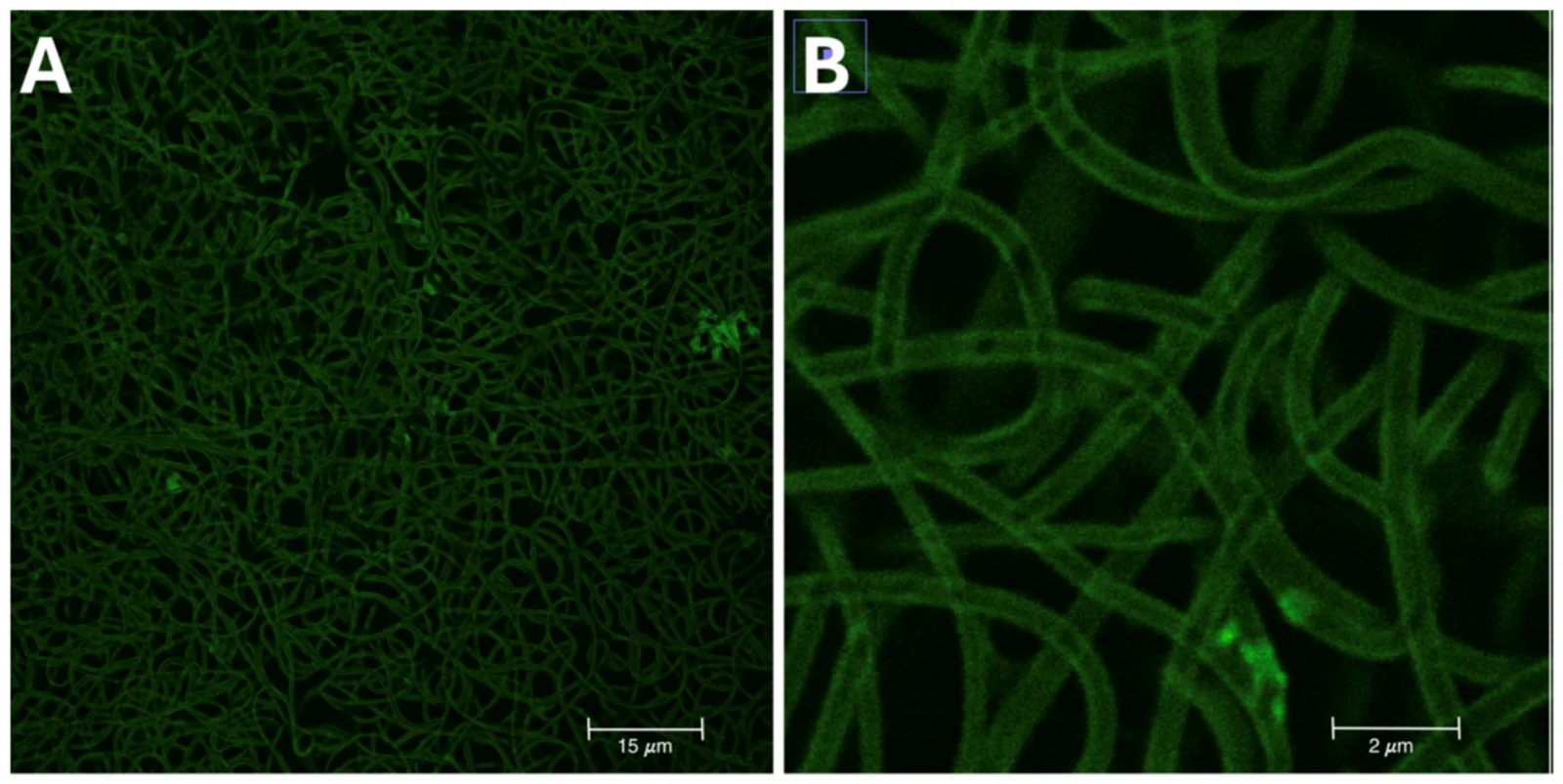
Collagen surface functionalization of electrospun PCL nanofibers. Confocal microscopy images of FITC-labeled type I collagen showing its distribution along the electrospun fibrous network at (**A**) lower and (**B**) higher magnification. Reprinted from Iurilli et al. [65] under CC BY 4.0.

Collagen and gelatin are widely investigated because their chemistry derives from extracellular matrix proteins and can support cell–material interactions. In electrospinning, however, their behavior depends strongly on polymer concentration, solvent system, molecular conformation, and post-spinning stabilization. Their high-water affinity can lead to substantial swelling or loss of structural integrity in aqueous media if the fibrous material is not adequately stabilized. Collagen can also be introduced as an interfacial component rather than as the primary structural phase. For example, collagen-coated electrospun PCL nanofibers functionalized with silver nanoparticles showed a continuous fibrous morphology, fibroblast compatibility, and antibacterial activity [65]. In this configuration, PCL provided the structural fibrous phase, whereas collagen was localized at the material interface. The distribution of collagen along the electrospun PCL network is shown in Figure 4.

Chitosan is a cationic polysaccharide whose amino groups govern its pH-dependent solubility and contribute to mucoadhesive and antimicrobial interactions. Electrospinning of chitosan is nevertheless constrained by restricted solvent compatibility, strong inter- and intramolecular interactions, and the difficulty of obtaining solution properties suitable for stable fiber formation. Chitosan is therefore frequently blended with carrier polymers such as poly(ethylene oxide), poly(vinyl alcohol), PCL, or gelatin, or incorporated as a surface or functional component rather than used as the sole structural phase. An in vitro comparison of alginate- and chitosan-based electrospun nanofibers revealed differences in fluid absorption, release behavior, antibacterial activity, and cytocompatibility [66].

Hyaluronic acid and alginate are highly hydrophilic polysaccharides of particular interest for interfaces requiring substantial water uptake. Hyaluronic acid is a glycosaminoglycan containing carboxyl and hydroxyl groups that contribute to its high affinity for water, whereas alginate is an anionic polysaccharide whose carboxylate groups enable ionic crosslinking with multivalent cations. For electrospun materials, this hydrophilicity can be advantageous for hydration and fluid interaction but can also result in rapid swelling, dissolution, or loss of fibrous morphology. Stabilization is therefore required when structural persistence under aqueous or physiological conditions is needed. Citric-acid-crosslinked PVA/HA nanofibers provide a representative electrospun example in which a highly hydrophilic system was stabilized by solvent-free solid-state esterification while retaining an aqueous fabrication route [14]. The resulting material showed that chemical stabilization can be used to limit the loss of fibrous structure associated with prolonged aqueous exposure. Related studies on crosslinked alginate hydrogels and electrosprayed microspheres further illustrate how polysaccharide chemistry and crosslinking can be used to modulate hydration and responsive behavior [67,68,69]. Although these systems are not electrospun fibers, they support the broader materials-design principle that polysaccharide chemistry, crosslinking, and water uptake must be considered together when designing responsive biomedical materials.

Silk fibroin and cellulose derivatives provide additional routes for combining bio-derived chemistry with comparatively stable fibrous structures. Silk fibroin can provide favorable mechanical properties, but its processing and final behavior depend on dissolution, regeneration, molecular conformation, and the development of β-sheet-rich structures. Co-electrospun silk fibroin–collagen and chitosan–PVA systems incorporating vitamin D3 and cerium oxide nanoparticles have been investigated for diabetic wound healing, showing how natural polymer matrices can be combined with functional nanophases to obtain antioxidant and regenerative activity [70]. Cellulose derivatives, particularly cellulose acetate, provide improved solution processability compared with native cellulose and can be readily combined with inorganic phases. Cellulose acetate nanofibers containing bioactive glass nanoparticles showed antibacterial activity and accelerated wound closure in a diabetic rat model [71].

The main challenge in electrospinning natural and bio-derived polymers is to achieve reproducible fiber formation while preserving their intended interfacial or biological functions under physiological conditions. Strategies such as blending, crosslinking, and surface functionalization should therefore be selected according to the required balance between processability, wet-state stability, and biological performance. Increased hydrophilicity or short-term cytocompatibility alone does not demonstrate long-term functionality, which must also be evaluated in relation to structural integrity, mechanical-property retention, degradation, sterilization, and the experimental conditions used for biological validation.

### 3.3. Blends, Composites and Hybrid Systems

Polymer blends and composites are widely investigated to combine structural, physicochemical, and biological functions that may be difficult to achieve using a single polymer. Their properties depend not only on nominal composition but also on polymer compatibility, phase distribution, and interfacial interactions. Blending synthetic polymers with natural or bio-derived components can improve specific interfacial properties, although changes in solution viscosity, conductivity, crystallization, and phase separation may also affect jet stability, fiber morphology, and mechanical behavior [72].

Electrospun PCL/gelatin/arbutin membranes provide an example in which the polymer blend defines the structural matrix while the incorporated bioactive molecule introduces additional functionality [60]. Similarly, gelatin/chitosan/PCL nanofibers containing carbon quantum dots and citrate showed changes in porosity, hydrophilicity, degradation, antibacterial activity, and wound-healing-related performance as a function of the multicomponent formulation [73].

Composite electrospun fibers incorporate a dispersed secondary phase, commonly inorganic particles or nanomaterials, within the polymer matrix. Hydroxyapatite, bioactive glass, ZnO, TiO_2_, silver nanoparticles, carbon-based nanomaterials, and other fillers can introduce osteoconductive, antibacterial, photothermal, conductive, antioxidant, or ion-releasing functions. Their incorporation, however, also changes the properties of the spinning solution and can affect viscosity, conductivity, surface tension, jet stability, crystallization, and solvent evaporation. Particle size, concentration, dispersion, and polymer–particle interactions therefore influence both fiber formation and the final physicochemical properties of the composite material [26]. At high filler contents or under poor dispersion conditions, particle aggregation can generate local defects, heterogeneous fiber diameters, surface protrusions, or unstable jet behavior. Filler incorporation should therefore be optimized according to both functional performance and process stability rather than loading level alone. Similar formulation–structure relationships have also been investigated in non-biomedical electrospun composites. For instance, water-based PVA/WO_3_ nanofibers were fabricated with increasing inorganic filler contents, while thermal crosslinking with citric acid was used to improve their stability in aqueous environments [74].

**Figure 5:**
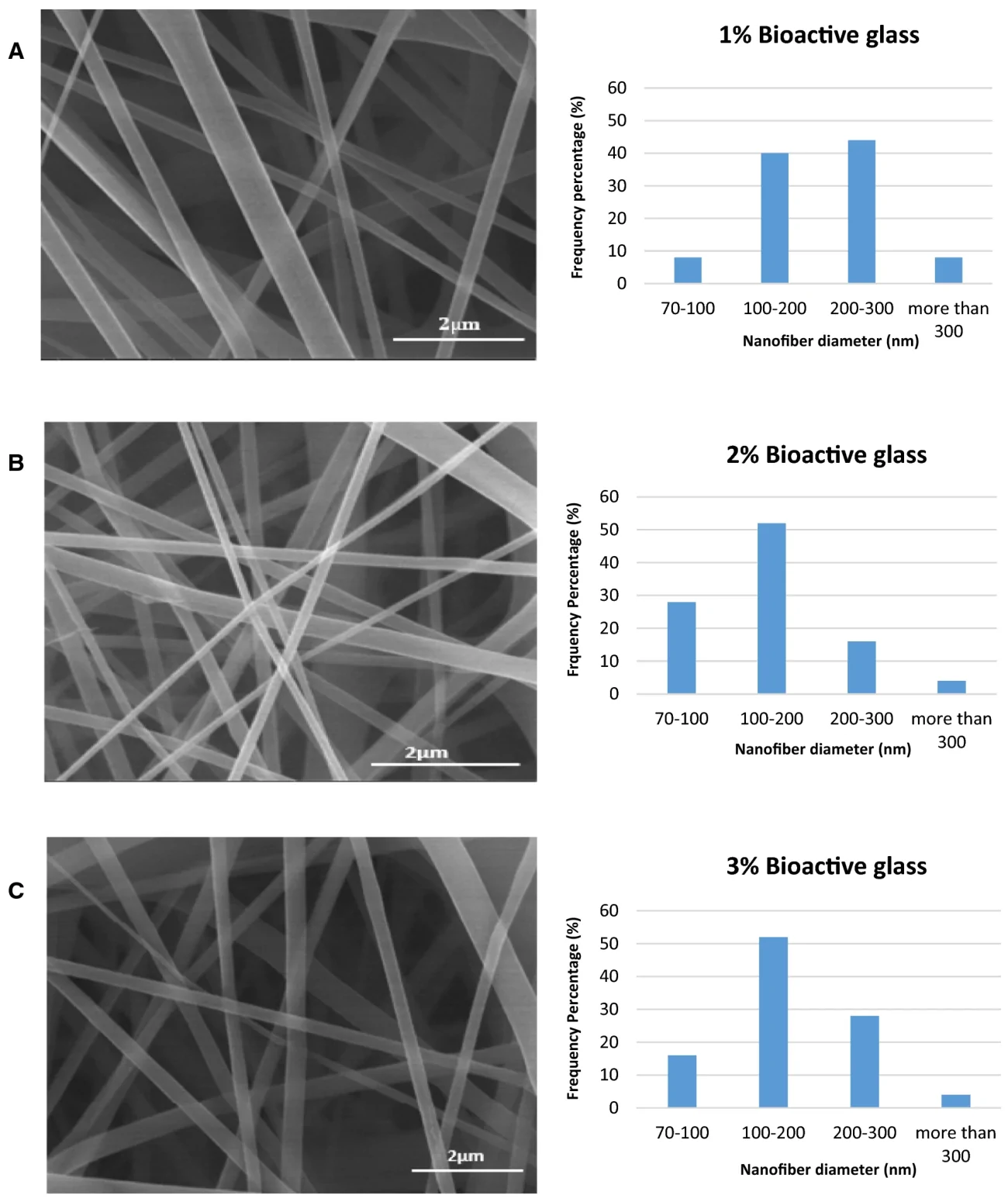
Effect of bioactive glass nanoparticle content on cellulose acetate electrospun nanofibers. SEM images and corresponding fiber-diameter distributions of nanofibers containing (**A**) 1%, (**B**) 2%, and (**C**) 3% bioactive glass nanoparticles. Reprinted from Sharaf et al. [71] under CC BY 4.0.

Cellulose acetate nanofibers containing increasing concentrations of bioactive glass nanoparticles showed changes in fiber-diameter distribution while maintaining a continuous fibrous morphology [71], as shown in Figure 5.

PLA/nano-hydroxyapatite coatings provide a relevant example of composite electrospun materials for titanium interfaces. In these systems, the PLA matrix provides the degradable fibrous phase, while nano-hydroxyapatite modifies the surface chemistry and biological response of the coating. Composition-dependent effects have been reported for fiber morphology, degradation and corrosion-related protection of the titanium substrate [25,61]. In wound-dressing applications, PLA/curcumin/TiO_2_ nanofibers and gelatin/chitosan/PCL systems containing carbon-based nanophases have also been investigated to combine structural support with antibacterial, antioxidant, or regenerative functions [73,75].

Increasing compositional complexity can be useful when different components are required to provide distinct structural or therapeutic functions. In these systems, polymer composition, bioactive-agent loading, and component distribution must be considered together because each phase can affect electrospinning behavior, degradation, release kinetics, and biological performance. Examples include PLGA-based fibers containing ZnO nanoparticles and VEGF [18], PLCL-based systems combining ZnO nanoparticles with cell-free fat extract [76], and PCL coatings incorporating BMP-2-loaded chitosan nanoparticles and α-tocopherol [77]. Table 2 summarizes representative polymer formulations, solvent systems, electrospinning and stabilization conditions, fiber architectures, and bioactive components. 

A major challenge in multicomponent electrospun materials is that each added component can affect both the electrospinning process and the properties of the resulting fibrous material. Nanoparticles can modify solution rheology, conductivity, particle dispersion, and jet stability, with subsequent effects on fiber morphology, crystallinity, surface roughness, and degradation. Hydrophilic polymers can increase water uptake and improve interfacial hydration but may also increase swelling and reduce wet-state mechanical stability. Similarly, crosslinking treatments can improve structural persistence while modifying stiffness, swelling, degradation, and biological response. Bioactive molecules may also lose activity during solvent exposure, electrospinning, drying, storage, sterilization, or subsequent hydration. Formulation and processing variables should therefore be evaluated as coupled parameters rather than optimized independently.

Compositional complexity should be introduced only when each component provides a defined structural, physicochemical, or biological function and the resulting material retains adequate processability and stability under application-relevant conditions. Rational formulation should identify which phase provides mechanical continuity, interfacial functionality, or therapeutic activity, while also considering phase distribution, bioactive-agent accessibility, release behavior, and stability during hydration, degradation, sterilization, and mechanical loading. Once composition has been defined, fiber architecture further controls the spatial localization, protection, and presentation of bioactive components.

## 4. Fiber Architecture and Bioactivation Strategies

The performance of bioactive electrospun materials depends not only on polymer composition but also on fiber organization and the spatial location of the bioactive function. Random and aligned fibers determine the organization of the fibrous network, whereas multilayer and core–shell configurations allow structural and functional components to be distributed across distinct regions of the material. These architectures can affect cell guidance, transport, payload accessibility, protection, and release.

**Figure 6:**
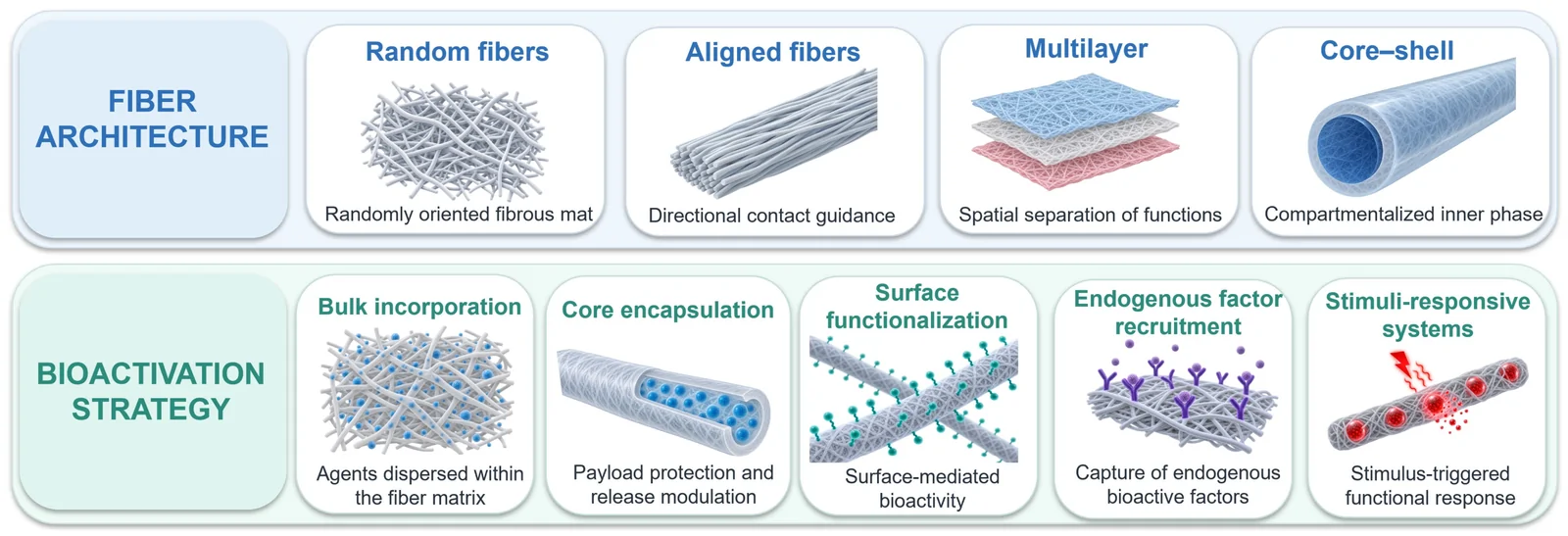
Main fiber architectures and bioactivation strategies in electrospun biomedical materials. Random, aligned, multilayer, and core–shell configurations can be combined with bulk loading, surface functionalization, endogenous-factor recruitment, and stimulus-responsive activation.

The location of the bioactive component is therefore an additional design variable. An active agent can be dispersed within the polymer matrix, confined within the core of a fiber, immobilized at the fiber surface, or recruited from the surrounding biological environment. These configurations differ in accessibility, transport mechanisms, and functional lifetime at the material–tissue interface. The main fiber architectures and bioactivation strategies considered in this review are summarized in Figure 6.

### 4.1. Architecture as Spatial Organization

Random electrospun mats provide a high interfacial area and an interconnected pore network without preferential in-plane fiber orientation. This configuration is suitable when conformability, surface coverage, fluid transport, or local delivery are primary requirements. In contrast, aligned fibers introduce anisotropic topographical and mechanical cues that can influence cell orientation and extracellular matrix organization. Aligned electrospun polyurethane nerve-guidance conduits, for example, have been combined with electrical functionality to modulate macrophage response and support peripheral nerve regeneration [87].

Multilayer constructs provide a means of assigning different functions to distinct regions of the material. Individual layers can be designed to contribute mechanical support, barrier properties, transport control, or biological activity, while their interfaces determine the integrity of the assembled construct. This approach is particularly relevant when the target tissue presents a stratified organization or when conflicting material requirements cannot be achieved within a single layer. Zhao et al. developed a bladder-regeneration scaffold combining electrospun PCL layers with an oxidized dextran/carboxymethyl chitosan hydrogel containing stem-cell-recruiting and VEGF-mimicking peptides [22]. The PCL layers provided mechanical reinforcement and reduced premature peptide loss, whereas the hydrogel phase provided the hydrated bioactive compartment.

Core–shell fibers provide radial separation of different material or functional phases within an individual fiber. In coaxial electrospinning, the shell can act as a diffusion-controlling layer and reduce direct exposure of the core phase to the surrounding environment, while the core can contain hydrophilic, rapidly diffusing, or labile bioactive agents. Release behavior depends on shell composition and thickness, core–shell compatibility, fiber diameter, polymer degradation, and payload–polymer interactions. Core–shell architectures can therefore reduce burst release and extend payload availability, although these effects must be demonstrated for each formulation rather than assumed from morphology alone [13,56,57]. Core–shell nanofibers containing epidermal-growth-factor-loaded nanoparticles and ciprofloxacin have been investigated for chronic wound healing, with the two therapeutic components selected to provide antibacterial and regenerative functions with different temporal requirements [88].

Taken together, these architectures present distinct advantages and limitations rather than a general hierarchy of performance. Fiber orientation and functional compartmentalization represent different design variables that may also be combined within the same construct. Randomly oriented mats generally provide a more directionally balanced mechanical response, whereas aligned fibers can promote directional reinforcement and contact guidance, although their resistance to transverse loading may be reduced. For example, Meng et al. [89] reported preferential osteoblast elongation along aligned PLGA/gelatin fibers, while cell numbers remained comparable to those observed on randomly oriented scaffolds. Fiber alignment can also influence mass transport by modifying network organization and diffusion pathways, although its effects depend on fiber packing, pore connectivity, membrane thickness, and material composition. Patrojanasophon et al. [90] observed faster α-arbutin release from highly aligned cellulose acetate nanofibers than from randomly oriented fibers. Conversely, Eslamian et al. [91] reported a lower initial burst release of dexamethasone from aligned PLGA fibers compared with randomly oriented counterparts. These findings indicate that the influence of fiber alignment on release behavior cannot be generalized independently of polymer–payload interactions and experimental conditions.

Multilayer and core–shell architectures provide different approaches to spatial compartmentalization. Multilayer systems enable the combination of structural, barrier, and bioactive functions within distinct layers, potentially allowing differentiated transport and release behavior, although additional interfaces may introduce limitations related to adhesion and interlayer transport [22]. Core–shell fibers instead provide radial separation of functional components within individual fibers, allowing the shell to regulate diffusion and protect the incorporated payload. However, their effectiveness depends on shell continuity, permeability, thickness, and degradation behavior [13,56,57]. Therefore, the selection of a specific architecture should consider not only its intended functional advantages but also the associated mechanical, transport, and manufacturing limitations, which should be evaluated under application-relevant conditions.

### 4.2. Localization of Bioactive Agents and Release Behavior

The choice among direct incorporation, core–shell localization, and surface functionalization depends on whether the intended function requires bulk loading, protection and controlled release of a payload, or direct presentation at the material–environment interface.

Direct incorporation of a bioactive agent into a single-fluid electrospinning solution is one of the simplest loading approaches, but the active component becomes part of the formulation and can alter viscosity, conductivity, polymer–solvent interactions, crystallization, and fiber morphology. Its distribution within the fiber is not necessarily homogeneous, since differences in solubility, diffusivity, and affinity for the polymer or solvent can promote preferential localization toward the fiber surface. Surface-enriched payloads can contribute to an initial burst release, whereas subsequent release may be governed by diffusion, polymer swelling, and degradation. The relative contribution of these mechanisms depends on both polymer chemistry and the physicochemical properties of the incorporated agent. When different bioactive functions require different exposure or release profiles, core–shell architectures may provide improved control over payload distribution. PLCL-based core–shell nanofibers incorporating zinc oxide nanoparticles and cell-free fat extract were investigated for antibacterial activity and their effects on fibroblast proliferation and migration in vitro [76]. In this case, fiber architecture was used together with material composition to control the presentation of components with distinct biological functions.

Control of localization is particularly relevant for proteins and growth factors because their biological activity can be affected by organic solvents, interfaces, dehydration, temperature, storage, and sterilization. Loading efficiency alone is therefore insufficient to evaluate these systems; retention of molecular activity after processing and during release should also be assessed. Core–shell loading, nanoparticle encapsulation, or surface immobilization can reduce some forms of exposure and provide additional control over presentation, but their effectiveness depends on the specific polymer–payload system. Release profiles should ultimately be matched to the time scale of the intended biological response, such as rapid infection control followed by sustained support of cell migration or angiogenesis.

### 4.3. Release Mechanisms and Transport Phenomena

Release from electrospun fibers involves payload dissolution and diffusion through a polymer matrix whose transport properties evolve during exposure. Surface-enriched or weakly associated agents can dissolve rapidly, producing an initial burst without polymer-chain degradation. Subsequent transport depends on payload solubility, polymer–payload interactions, matrix hydration and the continuity of diffusion pathways. Core–shell architectures introduce an additional barrier, whose effectiveness depends on shell thickness, permeability and stability. The sustained release reported by Zandi et al. from gelatin-based core–sheath fibers illustrates the contribution of spatial compartmentalization, although the release profile also reflects changes in the hydrated matrix [79].

Hydration can increase chain mobility and promote swelling, thereby changing effective diffusivity. In insufficiently stabilized hydrophilic fibers, dissolution and fiber fusion may eliminate the original architecture without requiring extensive chain scission. In hydrolytically degradable matrices such as PLGA, molecular-weight reduction and subsequent erosion progressively alter permeability and payload accessibility. Diffusion, swelling and degradation can therefore contribute concurrently to release rather than define separate sequential stages [92]. Morphological changes and mass loss alone do not distinguish these contributions; interpretation requires their relationship with water uptake and, where relevant, molecular-weight evolution.

Stimuli-responsive delivery couples transport to changes in polymer ionization, hydration, conformation or structural organization induced by pH, temperature or light [93]. Demonstrating this mechanism requires release measurements under defined stimulated and unstimulated conditions. Payload deactivation is a separate process: solvent exposure, interfacial adsorption, and dehydration can alter protein conformation, while temperature and local pH changes can promote denaturation or aggregation. Consequently, the amount released does not establish the amount remaining biologically active. Mechanistic interpretation requires release profiles to be considered alongside matrix evolution and payload stability, rather than inferred from curve fitting alone.

### 4.4. Interfacial and Responsive Bioactivation

Surface functionalization localizes the bioactive function at the fiber–environment interface rather than throughout the bulk polymer matrix. Because protein adsorption, cell attachment, and bacterial adhesion occur initially at this interface, changes in surface chemistry, charge, wettability, ligand density, and nanoscale topography can substantially affect biological interactions without requiring modification of the bulk structural phase. Plasma treatment, chemical grafting, coating, and biomolecule immobilization can therefore be used when the desired function depends primarily on interfacial presentation rather than sustained release.

An alternative strategy is to functionalize the fiber surface to recruit endogenous biological molecules rather than deliver exogenous factors. Casanova et al. developed a fibrous membrane designed to bind endogenous nerve growth factor (NGF) from urine and evaluated it after bilateral cavernous nerve injury [21]. The material was associated with improved nerve regeneration and functional outcomes compared with the control groups. This approach avoids direct loading of NGF but introduces other design requirements, including binding specificity, retention of surface functionality, variability in endogenous-factor availability, and stability of the functionalized interface.

Stimulus-responsive bioactivation introduces temporal control by coupling material function to an external trigger or to changes in the local environment. Temperature-responsive polymer systems, including PNIPAM-based composite hydrogels, have shown how a physicochemical transition can be coupled with additional functions such as electrical conductivity [94]. Although these systems are not electrospun fibers, the same materials-design principle can be applied to fibrous constructs by integrating responsive polymers or functional nanophases. In an electrospun wound dressing based on PLLA/quaternized chitosan nanofibers combined with hyaluronic acid, black phosphorus nanosheets, and hemoglobin, near-infrared irradiation generated a photothermal response through black phosphorus and promoted oxygen release from hemoglobin, while quaternized chitosan contributed antibacterial and hemostatic functions [19]. In this system, NIR irradiation provides an external means of controlling the photothermal response and promoting oxygen release from hemoglobin.

Increasing architectural complexity is justified only when spatial organization provides a measurable advantage over a simpler formulation. Additional layers, interfaces, or functional phases can affect electrospinning reproducibility, wet-state stability, interfacial adhesion, payload release, sterilization response, and manufacturing consistency. Architectural design should therefore be evaluated according to the localization and accessibility of the bioactive function, the stability of the assembled structure, and the ability to retain the intended function under application-relevant conditions. These requirements become application-specific in wound repair, urological regeneration, and implant coatings, as discussed in the following section.

The incorporation of a bioactive component should be distinguished from the experimental demonstration of its biological function. Confirmation of agent loading, surface localization, or release provides information on material composition and payload availability but does not necessarily establish the retention of biological activity or the occurrence of a therapeutic response. Functional claims should therefore be supported by assays appropriate to the proposed mechanism, using suitable controls to distinguish the effects of the incorporated agent from those of the polymer matrix. For stimulus-responsive systems, comparison between activated and non-activated conditions is also necessary to establish stimulus-dependent functionality. For example, the cytocompatibility observed for UV-C-treated PVA/HA nanofibers [95] supports their compatibility with the investigated cell models, but does not demonstrate an HA-specific regenerative effect. In contrast, antibacterial growth-inhibition assays and fibroblast proliferation and migration measurements performed on PLCL-based core–shell fibers containing ZnO and cell-free fat extract provide functional in vitro evidence under the investigated conditions [76], without necessarily establishing the individual contribution of each component. Animal studies reporting histological evidence of wound repair provide a further level of preclinical validation, although these findings cannot be directly extrapolated to clinical efficacy [73]. Biological claims should therefore reflect the specific endpoints measured and the level of experimental evidence available.

### 4.5. Electrospun Interfaces for Cell Sheet Engineering

Cell sheet engineering involves the formation and harvesting of cohesive cellular layers while preserving cell–cell junctions and cell-derived extracellular matrix. Culture substrates must therefore support tissue organization during growth and permit subsequent release without disrupting the cellular construct. Recent reviews identify harvesting efficiency, sheet mechanics and tissue-level function as central considerations for the development of these interfaces [96,97]. Electrospun substrates are relevant within this framework because fiber alignment and responsive polymer chemistry can be combined to regulate cellular organization and release.

Allen et al. investigated aligned poly(N-isopropylacrylamide)/polycaprolactone (PNIPAAm/PCL) fibers and obtained detached, aligned fibroblast sheets from the formulation containing 90% PNIPAAm [98]. PCL supported cell attachment and spreading, whereas cooling induced PNIPAAm dissolution and sheet release. The composition dependence of this response illustrates the balance between substrate persistence during culture and disruption during harvesting. Residual PCL was visible in the recovered sheets, indicating that successful detachment did not yield a completely polymer-free construct.

Karimipour et al. demonstrated light-controlled attachment and detachment of C6 glioma cells on spiropyran-functionalized acrylic nanofibers, associated with photoinduced changes in surface wettability [99]. These results demonstrate light-dependent cell attachment and detachment under the investigated conditions, whereas cohesive sheet harvesting represents a distinct functional endpoint. Across these approaches, the principal design challenge is to reduce cell–substrate adhesion sufficiently for release while preserving the organization and mechanical integrity of the cellular layer. Fiber-guided alignment and stimulus responsiveness therefore become relevant to cell sheet engineering when they support the recovery and subsequent handling of a cohesive tissue construct.

## 5. Biomedical Applications

The performance of bioactive electrospun materials must be evaluated against the physical, chemical, and biological conditions of the intended biomedical interface. Wound dressings, urological constructs, and implant coatings can exploit similar material features, including high surface area, interconnected porosity, and local bioactivation, but operate under markedly different conditions. Wound-contact materials are exposed to exudate, mechanical handling, and potential microbial contamination; urological constructs must function under persistent hydration, urine exposure, and repeated deformation; and implant coatings must remain integrated with a solid substrate while interacting with surrounding tissues and, depending on the application, bacterial biofilms or corrosive environments. These differences determine the relevant material requirements and failure mechanisms. Polymer composition, fiber architecture, degradation, wet-state mechanical behavior, payload release, and interfacial stability should therefore be evaluated according to the intended use rather than through a common set of performance criteria. The main application-specific requirements and validation endpoints are summarized in Table 3.

### 5.1. Wound Dressings and Skin Regeneration

Wound dressings represent a major application of bioactive electrospun materials because the wound bed is a dynamic interface characterized by continuous fluid exchange, mechanical handling, and, particularly in chronic wounds, persistent inflammation, microbial colonization, oxidative stress, impaired vascularization, and local hypoxia. The material must therefore manage wound fluids, retain sufficient structural stability, and preserve any incorporated antimicrobial or regenerative function.

Electrospun membranes can provide conformability, interconnected porosity, and a high surface area for interaction with wound fluids and for local presentation or release of bioactive agents. These characteristics alone, however, do not define an effective wound dressing. The fibrous material should retain its integrity under hydrated conditions, manage fluid uptake without excessive swelling or dissolution, and preserve the mechanical properties required for handling and removal. Test-medium composition should also be considered, since fluid-handling measurements obtained using simple saline solutions may not reproduce the behavior observed in protein-containing fluids that more closely represent wound exudate [106]. When therapeutic agents are incorporated, their release profile and biological activity must also remain compatible with the time scale of wound healing and with cytocompatibility requirements [100].

Multicomponent electrospun dressings have been developed to address several features of chronic wounds within the same material. Wang et al. integrated dual ZIF-based nanoplatforms within electrospun PCL/gelatin membranes for diabetic wound healing [107]. The fibrous matrix provided the structural dressing phase, whereas the incorporated ZIF-based components were designed to introduce antibacterial and pro-regenerative functions with controlled therapeutic delivery.

**Figure 7:**
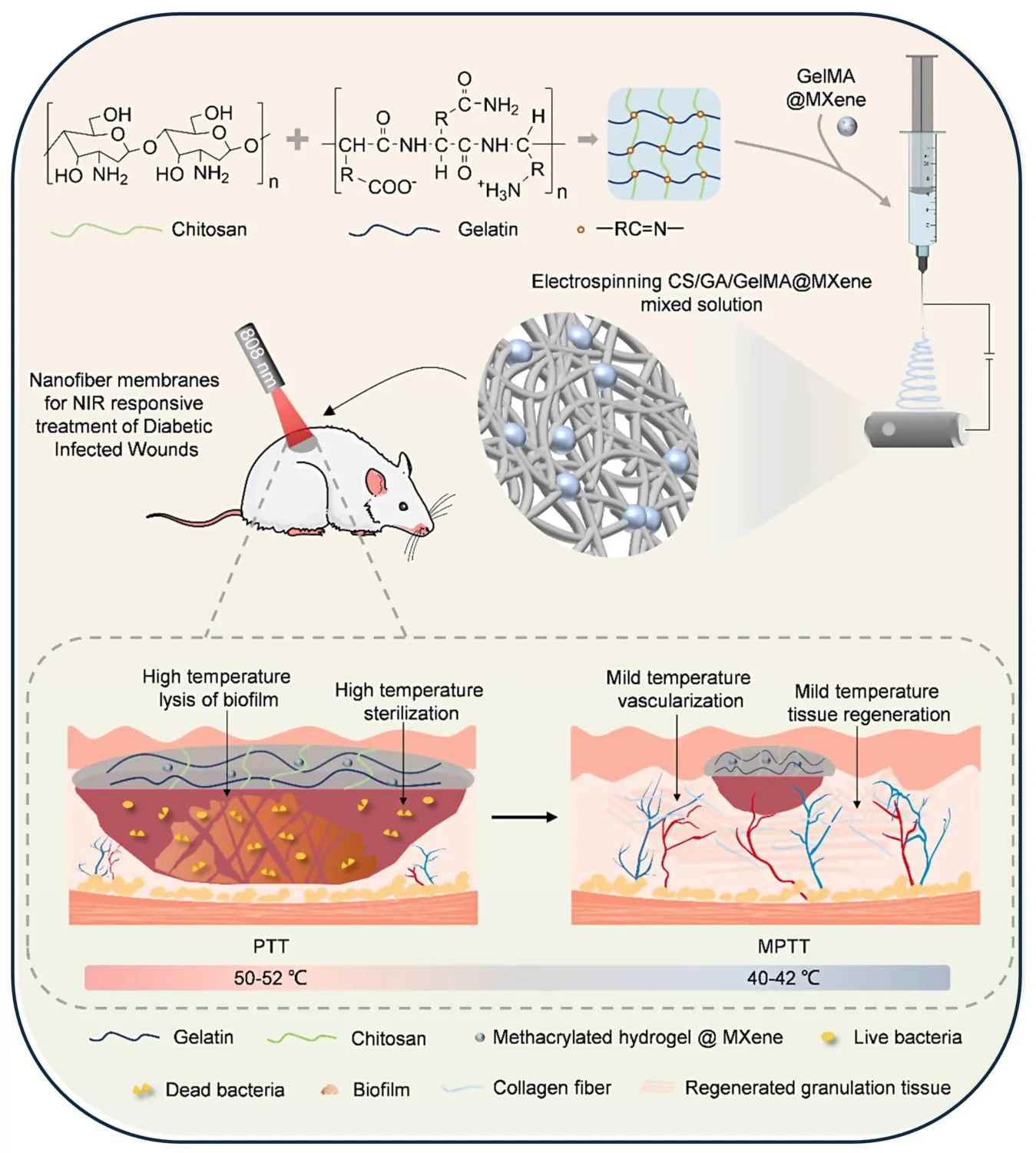
NIR-responsive electrospun nanofiber membrane for diabetic infected wound healing. Gel-MA@MXene-containing chitosan/gelatin nanofibers incorporating GelMA@MXene for temperature-dependent photothermal treatment, combining high-temperature antibacterial and antibiofilm activity (PTT, 50–52 °C) with mild-temperature stimulation of vascularization and tissue regeneration (MPTT, 40–42 °C). Reprinted from Fu et al. [101] under CC BY 4.0.

External activation provides an additional strategy for controlling wound-dressing function after application. Fu et al. incorporated GelMA@MXene microgels into electrospun chitosan/gelatin nanofibers for near-infrared-responsive treatment of diabetic infected wounds [101]. Higher photothermal temperatures were used for antibacterial and antibiofilm treatment, whereas milder temperature conditions were associated with regenerative responses, including vascularization and tissue repair. The MXene-containing phase therefore provided an externally addressable photothermal component whose response could be modulated through irradiation conditions. The temperature-dependent strategy is shown in Figure 7.

Oxygen delivery addresses a different limitation of chronic and diabetic wounds, where local hypoxia can impair tissue repair. Zhao et al. developed electrospun PLLA/quaternized chitosan nanofibers combined with hyaluronic acid, black phosphorus nanosheets, and hemoglobin [19]. Under NIR irradiation, black phosphorus provided the photothermal response and promoted oxygen release from hemoglobin, while quaternized chitosan contributed antibacterial and hemostatic functions. The material therefore combined oxygen delivery, photothermal activation, and antibacterial functionality within the same fibrous dressing.

**Figure 8:**
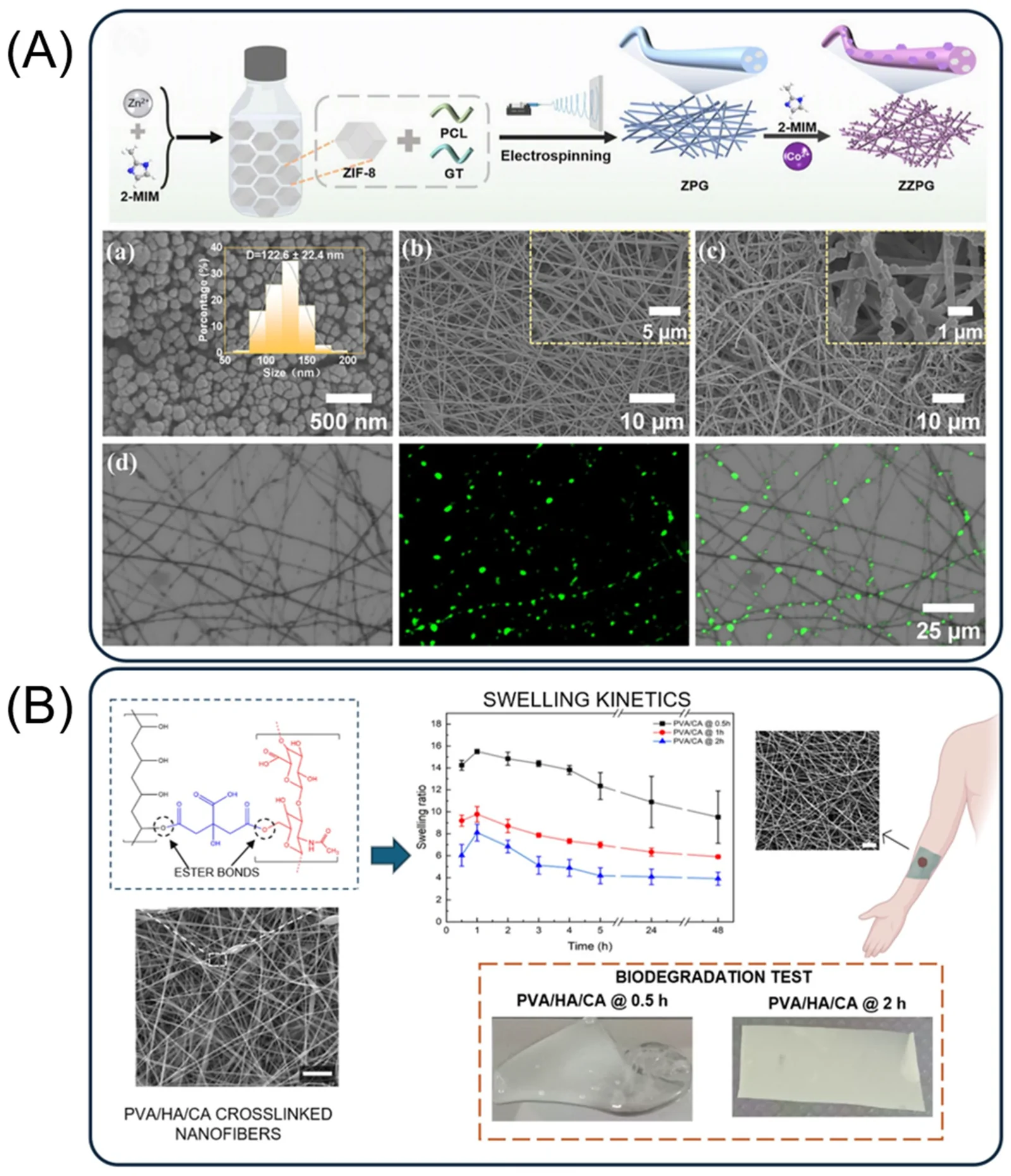
Complementary materials-design strategies for electrospun wound dressings. (**A**) Hierarchically organized PCL/gelatin nanofibers integrating ZIF-based nanoplatforms for spatiotemporally regulated therapeutic delivery. The subpanels show (**a**) ZIF-8 nanoparticles, (**b**,**c**) the morphology of electrospun fibrous structures, and (**d**) microscopy images of the composite fibrous system. Scale bars: (**a**) 500 nm; (**b**) 10 µm and 5 µm (inset); (**c**) 10 µm and 1 µm (inset); (**d**) 25 µm. Reprinted from Wang et al. [107] with permission from Wiley-VCH GmbH. © 2025 Wiley-VCH GmbH. (**B**) Citric-acid-crosslinked PVA/HA nanofibers illustrating the esterification mechanism, swelling kinetics, fibrous morphology, and structural stability following aqueous exposure. The SEM images in panel B include scale bars of 3 µm. Reprinted from Ciarleglio et al. [14] under the Creative Commons Attribution 4.0 International License (CC BY 4.0).

Wet-state stability represents a separate and fundamental design requirement. Highly hydrophilic polymers such as PVA, hyaluronic acid, alginate, and gelatin can promote water uptake and interaction with wound fluids, but excessive swelling, dissolution, or loss of fibrous morphology may compromise handling, transport properties, and functional persistence. Chemical or physical stabilization is therefore required when the fibrous structure must be retained during prolonged aqueous exposure. Citric-acid-crosslinked PVA/HA nanofibers provide an example in which solvent-free solid-state esterification was used to stabilize a highly hydrophilic electrospun system while maintaining an aqueous fabrication route [14]. The effects of chemical stabilization on swelling and structural persistence are shown in Figure 8B.

The main challenge for electrospun wound dressings is therefore not the incorporation of multiple functions per se, but their retention within a hydrated, protein-rich, mechanically disturbed, and potentially infected wound environment. Cross-study comparison is further limited by differences in polymer composition, mat thickness, crosslinking conditions, fluid-exposure protocols, release duration, microbial strains, cell models, and in vivo wound models. Favorable results obtained for swelling, antimicrobial activity, or short-term cytocompatibility should therefore not be considered equivalent evidence of therapeutic performance. Future studies should evaluate fluid uptake, wet-state structural stability, release behavior, antimicrobial activity, cytocompatibility, and regenerative response under application-relevant conditions. In multifunctional or stimulus-responsive systems, the contribution of individual components and activation mechanisms should also be resolved experimentally to determine whether the added material complexity results in a measurable functional advantage.

### 5.2. Urological Repair and Regeneration

Urological applications impose highly tissue-specific requirements on electrospun materials, reflecting the distinct structural, mechanical, barrier, and regenerative functions of the different tissues of the urinary system. Bladder and urethral constructs must retain structural and mechanical function under persistent hydration, urine exposure, and repeated deformation, while also supporting tissue-specific regeneration [20,102,103,108]. Materials intended for cavernous nerve repair are subjected to different requirements, including maintenance of a permissive interface for neural regeneration and control of fibrosis. Electrospun architectures should therefore be selected according to the mechanical, transport, and biological functions required by the target urological tissue rather than treated as a common scaffold configuration.

Bladder reconstruction is particularly demanding because the material must accommodate repeated filling–emptying deformation while maintaining adequate compliance and supporting restoration of urothelial, stromal, vascular, and smooth-muscle components [109]. Multilayer architectures are attractive in this context because mechanically and biologically distinct functions can be distributed across separate material regions. Zhao et al. developed a biomimetic scaffold combining electrospun PCL nanofibrous layers with an oxidized dextran/carboxymethyl chitosan hydrogel containing stem-cell-recruiting and VEGF-mimicking peptides [22]. The PCL layers provided mechanical reinforcement and contributed to peptide retention, whereas the hydrogel phase provided a hydrated environment for presentation of the bioactive peptides. In vivo, the combined construct was associated with improved bladder regeneration, smooth-muscle recovery, innervation, and reduced fibrosis.

A different bladder-regeneration strategy was reported by Wang et al., who combined electrospun silk fibroin with extracellular matrix derived from adipose-derived stem-cell sheets and VEGF-conjugated Fe_3_O_4_ nanoparticles [110]. In this system, the electrospun fibrous phase provided structural support, while the ECM-derived and nanoparticle-associated components supplied additional biological cues related to cell interaction and vascularization.

**Figure 9:**
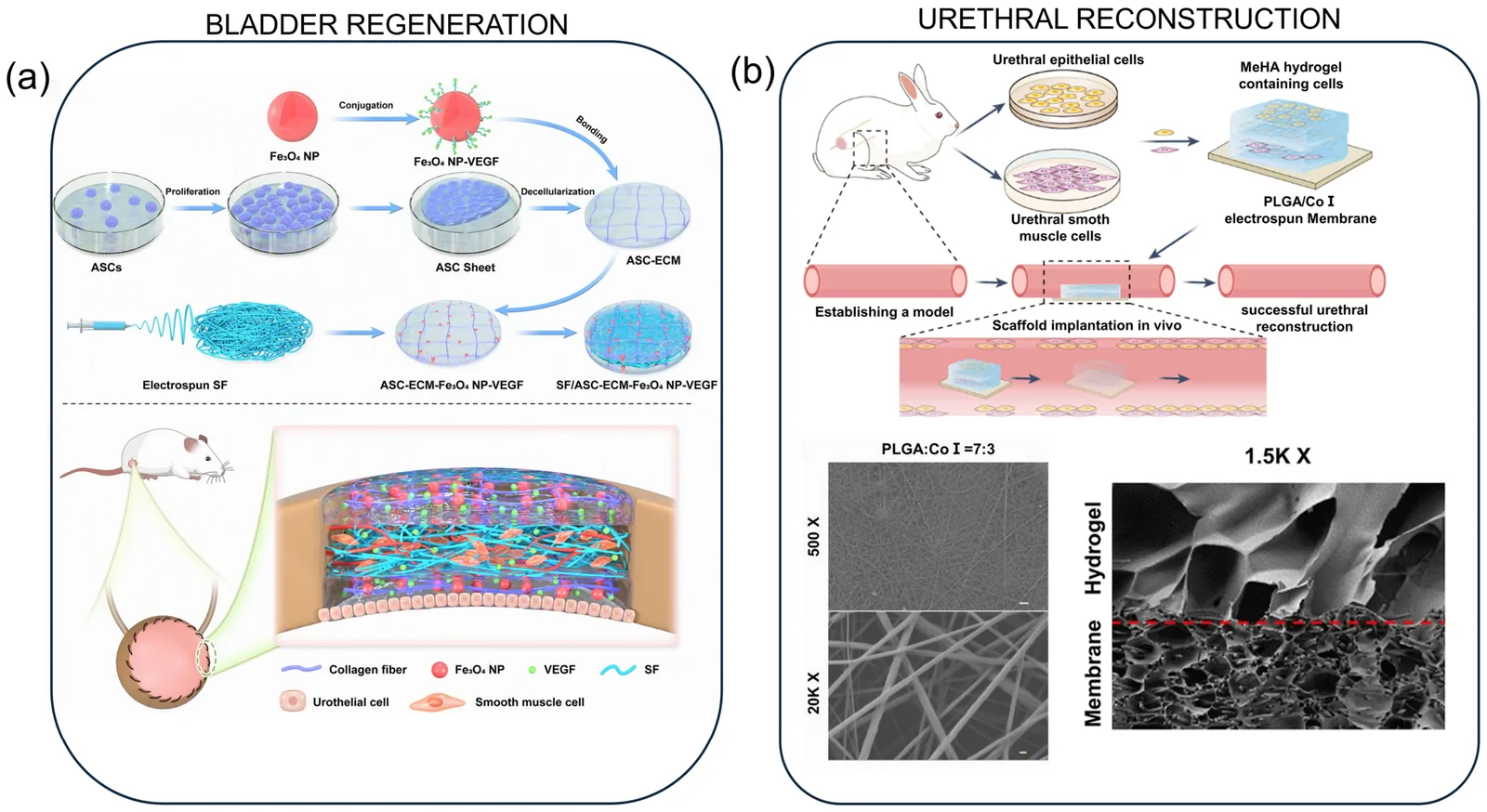
Electrospun hybrid constructs for lower urinary tract regeneration. (**a**) Silk-fibroin/ASC-derived ECM construct functionalized with VEGF-conjugated Fe_3_O_4_ nanoparticles for bladder regeneration. Reprinted from Wang et al. [110] under CC BY 4.0. (**b**) Double-layer PLGA/CoI-MeHA tissue-engineering scaffold for urethral reconstruction, combining an electrospun PLGA/CoI membrane with photocrosslinked MeHA hydrogel. SEM images show the morphology of the PLGA/CoI (7:3) electrospun membrane at 500× and 20,000× magnification and the membrane–hydrogel interface at 1500× magnification. Reprinted from Chang et al. [82] under CC BY 4.0.

Urethral reconstruction requires a different balance between structural support, compliance, fluid exposure, and surgical handling. Chang et al. developed a double-layer construct combining an electrospun PLGA/collagen type I (PLGA/CoI) membrane with a methacrylated hyaluronic acid (MeHA) hydrogel [82]. The electrospun layer provided structural support, while the hydrogel phase provided a hydrated cell-supportive compartment. Such combinations are relevant when neither a dense polymeric layer nor a highly hydrated hydrogel alone can provide the required balance between mechanical integrity and biological interaction. Representative bladder- and urethra-regeneration strategies are shown in Figure 9.

Surgical handling is often insufficiently addressed when characterizing an electrospun scaffold. Sturm et al. developed electrospun constructs for lower urinary tract reconstruction with emphasis on suturability and extensibility [80]. These results emphasize that fibrous morphology and cytocompatibility alone are insufficient for surgical reconstruction when suturability and deformation resistance are required.

Electrospun fibrous materials have also been investigated for post-surgical cavernous nerve repair. Casanova et al. developed a cell-free fibrous membrane functionalized to recruit endogenous nerve growth factor (NGF) from urine and evaluated it in a rat model of bilateral cavernous nerve crush injury [21]. The membrane was associated with improved nerve regeneration and functional outcomes, together with favorable neural, vascular, and fibrosis-related markers compared with the control groups. Unlike conventional growth-factor-loaded systems, the bioactive function depended on surface-mediated recruitment of an endogenous factor rather than the release of a preloaded therapeutic agent.

Urological applications therefore require electrospun materials to be evaluated as hydrated and mechanically active constructs rather than as dry fibrous scaffolds. Relevant validation should reflect the target tissue and may include wet-state mechanical behavior, degradation in urine-relevant media, dimensional and morphological stability, surgical handling and suture retention, barrier formation, smooth-muscle remodeling, innervation, fibrosis, and long-term functional recovery. The required endpoints differ between bladder, urethral, and nerve-repair applications, and material performance should therefore be assessed against the dominant failure mechanisms of each tissue interface.

Compared with wound-healing applications, the evidence base for electrospun urological materials remains less extensive and more heterogeneous. Many studies report morphological characterization, short-term cytocompatibility, or initial regenerative responses, whereas fewer assess prolonged urine exposure, cyclic deformation, barrier restoration, infection-related conditions, suturability, or long-term tissue-specific function. This heterogeneity currently limits the definition of robust materials-selection criteria for bladder, urethral, and nerve-repair applications. A relevant priority is therefore the demonstration that structural and functional properties are retained under urine-relevant chemical conditions and mechanically representative loading over time scales compatible with tissue repair.

### 5.3. Implant and Biomedical Device Coatings

Implant and biomedical device coatings represent a distinct application of bioactive electrospun materials because the fibrous layer is deposited onto a pre-existing solid substrate rather than used as a free-standing construct. The coating must therefore provide the intended biological or physicochemical function while maintaining sufficient adhesion, structural integrity, and stability at the substrate interface. Depending on the device, relevant functions may include tissue integration, local therapeutic delivery, antibacterial activity, and, for metallic substrates, corrosion-related protection.

Titanium and titanium alloys are widely used in dental and orthopedic devices because of their mechanical properties and corrosion resistance. Electrospun coatings can introduce a porous polymeric layer containing bioactive molecules, ceramic phases, or antimicrobial agents when additional interfacial functions are required. Their performance, however, should be evaluated within a coupled substrate–coating–tissue system, since favorable biological activity is of limited relevance if the fibrous layer undergoes delamination, cracking, premature degradation, or loss of function during use.

Electrospun coatings can function as local delivery interfaces for osteogenic stimulation. Karataş et al. developed titanium dental implants coated with PCL nanofibers containing BMP-2-loaded chitosan nanoparticles and α-tocopherol [77]. In this system, PCL provided the degradable fibrous matrix, while the incorporated components introduced osteogenic and antioxidant functions. The coating showed prolonged BMP-2 release together with an osteogenic response, supporting the use of electrospun fibers as local therapeutic interfaces rather than solely as topographical surface modifications.

A complementary strategy involves the incorporation of mineral bioactive phases within the electrospun coating. PLA/nano-hydroxyapatite coatings have been directly deposited onto titanium using a green-solvent electrospinning approach, producing nanofibrous layers with composition-dependent morphology and degradation behavior [61]. Related PLA/nano-hydroxyapatite coatings were also investigated for improved biocompatibility at the titanium interface [62] and for corrosion protection of titanium substrates [25]. The contribution of the inorganic phase to biological response and corrosion-related behavior must therefore be interpreted together with coating morphology, degradation, and substrate interaction.

**Figure 10:**
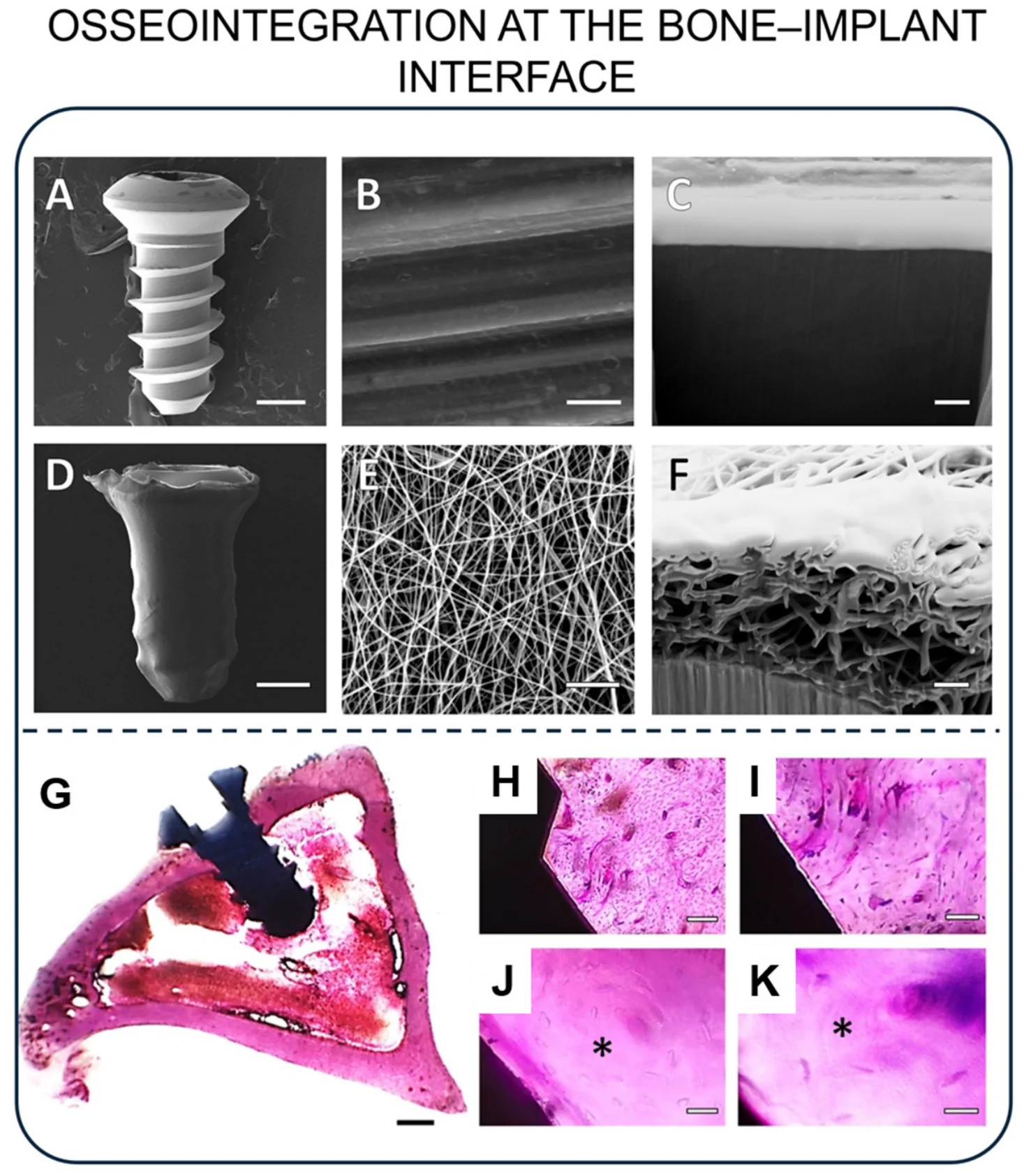
Osteogenic electrospun coatings for bone–implant integration. (**A**–**C**) SEM images of uncoated titanium implants showing implant morphology, surface topography, and cross-sectional structure, respectively. (**D**–**F**) Corresponding SEM images of titanium implants coated with PCL/gelatin/hydroxyapatite-based nanofibers, showing coating morphology and fibrous architecture. Scale bars: (**A**,**D**) 1 mm; (**B**,**E**) 5 µm; (**C**,**F**) 1 µm. (**G**–**K**) Representative hematoxylin and eosin (H&E)-stained histological sections of coated implants after 12 weeks in a rabbit tibial model, showing bone–implant integration at different magnifications. Asterisks (*) indicate mature bone in contact with the implant surface. Scale bars: (**G**) 1 mm; (**H**) 100 µm; (**I**) 50 µm; (**J**) 20 µm; (**K**) 10 µm. Reprinted from Das et al. [85] under CC BY 4.0.

Tissue integration can also be promoted through multicomponent osteogenic coatings. Das et al. deposited an electrospun composite containing PCL, gelatin, hydroxyapatite, and osteogenic additives onto titanium screw implants [85]. In a rabbit tibial model, coated implants showed improved peri-implant bone formation and osseointegration-related outcomes compared with uncoated controls. The coating morphology and representative peri-implant response are shown in Figure 10.

Electrospun surface engineering can also target soft-tissue integration at transmucosal interfaces. Ferraris et al. combined mechanically generated titanium nanogrooves with electrospun keratin nanofibers and evaluated their interaction with gingival fibroblasts [111]. Nanogrooved surfaces promoted directional fibroblast organization, while the keratin nanofibrous component enhanced fibroblast adhesion and proliferation. The investigated surface modifications did not increase *Staphylococcus aureus* adhesion relative to polished titanium. These results support concurrent evaluation of soft-tissue integration and bacterial adhesion when designing transmucosal implant surfaces.

Bacterial colonization and biofilm formation represent additional failure mechanisms for implantable devices. Lashkarara et al. combined a plasma-electrolytic-oxidized Mg_3_ZnCa substrate with electrospun cefiderocol-loaded PCL/gelatin nanofibers and bacteriophages [105]. In this system, substrate modification addressed corrosion-related performance, whereas the electrospun layer provided localized antibacterial functionality. The study therefore combined substrate protection and infection control within the same surface-engineering strategy.

**Figure 11:**
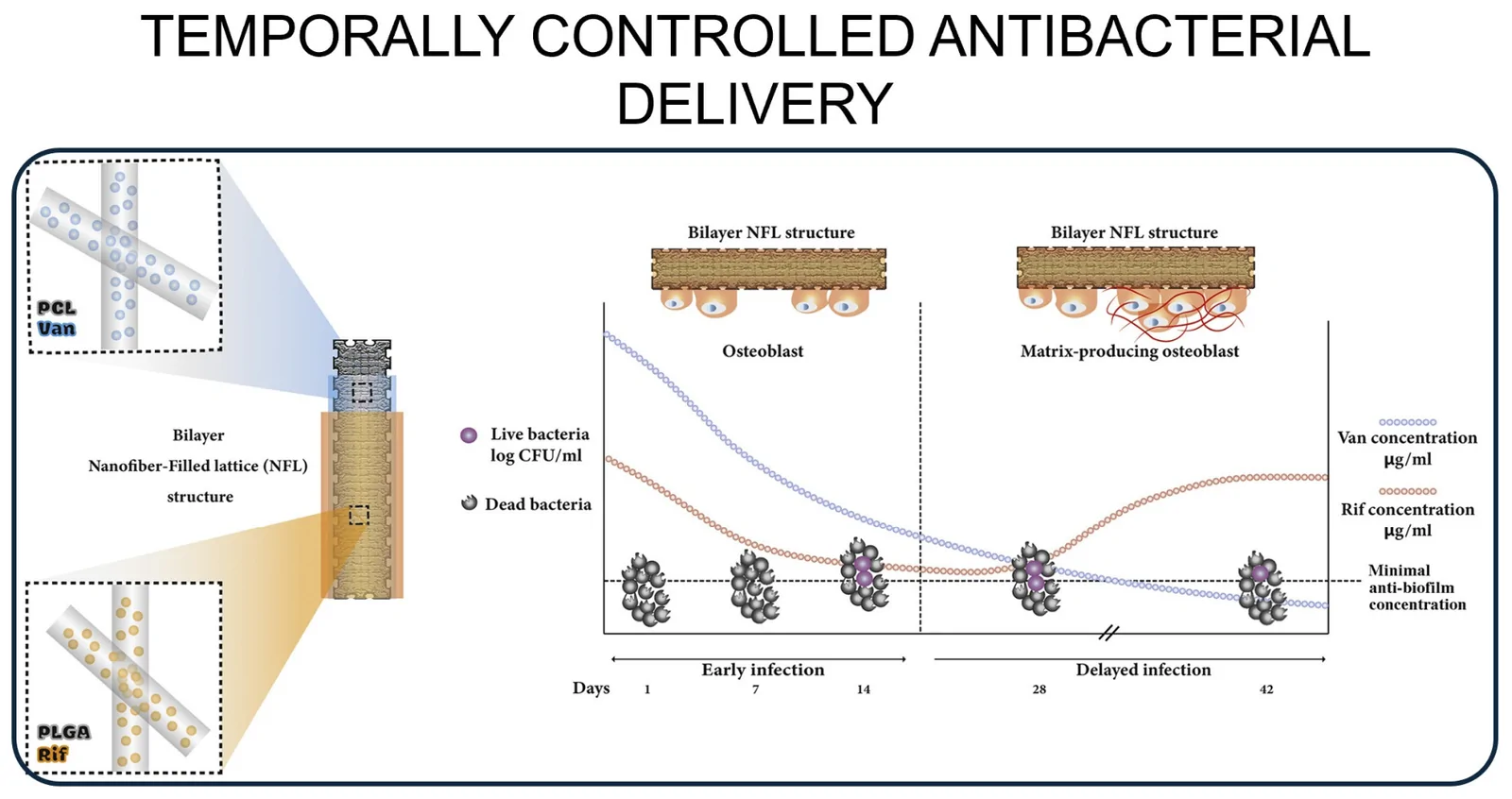
Bilayer electrospun nanofibers integrated within a titanium lattice for local antibiotic delivery. The construct combines an inner vancomycin-loaded PCL layer and an outer rifampicin-loaded PLGA layer to provide distinct antibiotic release profiles. Reprinted from Jahanmard et al. [84] under the Creative Commons Attribution 4.0 International License (CC BY 4.0).

Temporal control of antibacterial delivery can also be introduced through multilayer electrospun coatings. Jahanmard et al. developed a nanofiber-filled titanium lattice containing an inner vancomycin-loaded PCL layer and an outer rifampicin-loaded PLGA layer [84]. Spatial separation of the antibiotic-containing layers produced distinct release profiles, while incorporation of the nanofibrous assembly within the metallic lattice reduced the risk of delamination during press-fit implantation. This design combined therapeutic release control with mechanical protection of the coating. The bilayer nanofibrous architecture integrated within the titanium lattice is shown in Figure 11.

Electrospun implant and biomedical-device coatings should be evaluated in terms of their biological or therapeutic function and their integrity at the substrate interface. Cytocompatibility, antibacterial activity, therapeutic release, or tissue-integration potential are of limited relevance if the fibrous coating undergoes delamination, cracking, premature degradation, or loss of adhesion during implantation or physiological exposure. Relevant validation endpoints therefore include coating adhesion and delamination resistance, wet-state durability, sterilization stability, release under physiological conditions, antibacterial activity against adherent bacteria or biofilms, and tissue-specific integration. Corrosion behavior and wear resistance should additionally be considered when electrochemical stability or mechanical contact are relevant to the intended device. These requirements vary with the application: dental implants are exposed to combined mechanical and microbial challenges, orthopedic devices require durable bone integration and infection control, while biodegradable metallic substrates introduce additional constraints related to substrate degradation and corrosion. Electrospun coatings should therefore be considered within an integrated substrate-coating-tissue system in which adhesion, degradation, release behavior, and biological performance remain compatible with the intended use.

Table 4 compares the formulations with their measured outcomes, exposure conditions and biological models.

The comparison in Table 4 highlights the difficulty of balancing bioactive delivery with structural and interfacial persistence. Incorporating active components can modify hydration, polymer interactions, and fiber organization, coupling the intended biological function to changes in mechanical and transport properties. Higher loading or prolonged release therefore provides an incomplete measure of material performance unless considered over the period during which mechanical support, barrier function, or substrate attachment is required. This balance differs across applications: wound dressings require fluid management and handling integrity, urological constructs require compliance and tissue-barrier restoration, and implant coatings require durable substrate retention. However, these functions are often assessed separately and over different experimental timescales. The resulting evidence supports individual material attributes more convincingly than their coordinated preservation throughout the intended functional lifetime.

## 6. Discussion and Translational Outlook

The translational potential of bioactive electrospun materials depends on whether relationships established among formulation, processing, fiber structure, and bioactive function remain valid under the conditions imposed by the target biomedical interface. Wound dressings, urological constructs, and implant coatings share a fibrous morphology but differ substantially in hydration, mechanical loading, microbial exposure, functional lifetime, and mode of interaction with the surrounding tissue. Their performance should therefore be assessed against application-specific requirements rather than through a common set of morphological or biological descriptors.

A recurring limitation in electrospun biomaterials research is the reliance on dry-state morphology and short-term biological assays as primary indicators of performance. Fiber diameter, porosity, and defect-free morphology are important processing descriptors, but they do not establish whether the material will retain its structure and function after hydration, swelling, degradation, protein adsorption, or mechanical deformation. This distinction is particularly relevant for highly hydrophilic systems, in which exposure to aqueous media can substantially alter fiber morphology and mechanical behavior. Citric-acid-crosslinked PVA/HA nanofibers showed how chemical stabilization can improve structural persistence under prolonged aqueous exposure [14], while urethral constructs combining electrospun and hydrogel phases emphasize the need to evaluate mechanics and degradation under conditions relevant to the target tissue [82].

Wet-state behavior depends on the chemical and mechanical exposure conditions, as well as on hydration itself. In charged polymer networks, ionic screening modifies electrostatic interactions and osmotic swelling; Liu et al. reported decreasing swelling with increasing ionic strength in electrospun poly(acrylamide-co-(maleic acid)) hydrogel fibers [112]. Protein adsorption introduces a further change in the interface encountered by cells and dissolved components. Tanes et al. demonstrated time- and concentration-dependent albumin adsorption on electrospun PCL fibers, although this observation does not independently establish a change in bulk structural stability [113]. Medium-dependent changes in PCL crystallinity and fiber morphology were reported for PCL/gelatin blends exposed to water, PBS and culture medium [114]. Mechanical exposure can also alter the degradation trajectory: continuous tensile loading accelerated molecular-weight reduction and morphological disruption in electrospun PLGA scaffolds [115]. These findings support the evaluation of swelling, structural evolution and mechanical retention under defined medium compositions and loading histories. Static immersion provides a baseline, whereas its relevance to use depends on whether the selected conditions reproduce the dominant exposures of the target interface.

The evidence summarized in Table 4 addresses distinct aspects of material performance. In vitro assays characterize cellular responses and antibacterial activity, while ex vivo evaluations assess specific procedural requirements, such as coating retention during press-fit implant insertion [84]. Animal studies extend the assessment to tissue-level outcomes, including wound re-epithelialization [73], urethral regeneration [82] and bone–implant contact [85]. However, tissue formation and favorable histological findings provide limited information on the coordinated evolution of degradation, mechanical integrity and functional recovery. Follow-up duration, implantation site and loading conditions therefore remain essential to interpreting these results. The principal translational gap concerns the extent to which the available endpoints demonstrate preservation of the intended material function over time, particularly under the chemical, mechanical and biological conditions of the target interface.

Sterilization, storage, and manufacturing reproducibility should be considered part of material development rather than downstream operations [15,16,17]. Electrospun materials are sensitive to solution preparation, environmental conditions, deposition geometry, and post-processing, and variations in these parameters can modify fiber-diameter distribution, thickness, porosity, crystallinity, residual solvent content, mechanical behavior, degradation, and payload distribution. Sterilization and storage may further alter polymer properties or reduce the activity of incorporated biological agents. Consequently, morphology, wet-state mechanics, degradation, release behavior, and biological function should be assessed after the processing and storage steps expected for the final material.

Sterilization can affect electrospun materials even when their fibrous morphology appears unchanged. Gamma irradiation and electron-beam (e-beam) irradiation are low-temperature ionizing processes, but both may induce polymer-chain scission and changes in molecular weight, crystallinity, and subsequent degradation behavior. In electrospun PCL mats exposed to 25 and 33 kGy, de Cassan et al. [116] reported a reduction in molecular weight and an increase in crystallinity following irradiation, with e-beam producing smaller changes than gamma treatment, whereas mechanical properties were not significantly affected under the investigated conditions. However, this does not establish a general advantage of e-beam, since the effects of irradiation depend on polymer chemistry, absorbed dose, dose rate, and exposure conditions. Ethylene oxide (EtO) sterilization avoids high processing temperatures but introduces limitations related to gas residues and the need for adequate aeration. Its effects on fiber morphology are formulation-dependent: Horakova et al. [117] observed no substantial morphological changes in electrospun PCL following EtO treatment, whereas Valente et al. [118] reported loss of fiber alignment and the formation of ribbon-like structures in electrospun PLA membranes.

Steam autoclaving introduces different limitations because the combined effects of elevated temperature and moisture may induce fiber fusion, dimensional changes, hydrolytic degradation, or loss of structural integrity in susceptible polymer systems. Conventional steam sterilization is particularly unsuitable for electrospun PCL, whose melting temperature is considerably lower than the temperatures typically employed during autoclaving, while the compatibility of other polymer formulations must be established individually [119]. Beyond structural and physicochemical changes, sterilization may also compromise the stability of incorporated bioactive components. Preem et al. [120] demonstrated that sterilization of chloramphenicol-loaded electrospun matrices can affect polymer properties and drug stability even in the absence of evident morphological changes. Therefore, the selection of a sterilization method should consider not only the preservation of fiber morphology and polymer properties but also the retention of therapeutic activity and release behavior. Post-sterilization evaluation should include relevant physicochemical, mechanical, and biological characterization, together with validation of the sterilization process for the final material.

Scale-up introduces the same requirement for preservation of material attributes. Increasing throughput or deposition area can require changes in spinneret arrangement, solution handling, collector geometry, environmental control, and post-processing, which may modify the material even when the nominal formulation remains unchanged [16,17]. Scale-up should therefore be evaluated in terms of both production capacity and preservation of predefined material attributes. Batch-to-batch acceptance criteria should include the parameters that are functionally relevant to the intended application rather than morphology alone.

Multifunctionality introduces an additional design constraint because increasing the number of components also increases the number of possible interactions among formulation, processing, release, degradation, and biological response. Additional nanoparticles, proteins, antibiotics, or responsive elements should therefore be introduced only when they provide a measurable function that cannot be achieved by a simpler material. Appropriate controls are required to distinguish the contribution of individual components and to determine whether the combined formulation provides a reproducible functional advantage. From a translational perspective, increasing compositional complexity also increases the requirements for dose control, analytical characterization, stability assessment, sterilization, manufacturing reproducibility, and quality control.

Host responses to implanted electrospun materials depend on fiber organization and surface chemistry as well as polymer composition. Ma et al. reported a stronger foreign body response to electrospun yarn scaffolds than to aligned-fiber scaffolds, despite the directional organization of the yarn bundles, demonstrating that alignment alone does not determine the inflammatory outcome [121]. Surface bioactivation can likewise modify both inflammation and resorption. In electrospun PCL scaffolds, heparin immobilization prolonged the acute inflammatory phase, whereas subsequent platelet-lysate treatment reduced inflammation, slowed implant absorption and promoted vascularization [122]. Such responses must be interpreted in relation to implantation site and duration: reduced inflammation, delayed resorption and tissue ingrowth have different implications depending on the required support period. Macrophage responses, foreign body giant cells and fibrous encapsulation therefore complement cellular compatibility measurements when assessing integration and remodeling.

For products regulated as medical devices, development requires evaluation of the final formulation and manufacturing process in relation to intended use and tissue-contact duration. Regulation (EU) 2017/745 [123] provides the European regulatory framework, while ISO 14971:2019 [124] addresses risk management, ISO 10993-1:2025 [125] biological safety evaluation and ISO 10993-6:2026 [126] local effects after implantation. Relevant considerations include residual processing agents, degradation products, released particles and changes introduced by sterilization and storage. Biological safety assessment must be accompanied by longitudinal functional evidence: degradable constructs require sufficient support during tissue repair, whereas coatings require persistent substrate attachment and surface function. These requirements connect the evolution of the host response with the mechanical and interfacial stability of the final material.

Data-driven and machine-learning-assisted approaches may support application-driven materials engineering by integrating polymer chemistry, solution properties, processing parameters, fiber morphology, and functional outputs. Machine-learning models can support the identification of process–structure–property relationships across multivariable datasets. Recent studies and reviews have demonstrated the potential of these approaches for predicting fiber morphology and diameter, optimizing processing conditions, and supporting process monitoring and control in electrospinning [30,127,128,129]. At a broader biomaterials level, machine learning is increasingly being explored for candidate screening, property prediction, and experimental prioritization across complex composition–processing–structure–function spaces [31]. For bioactive electrospun materials, machine-learning approaches should extend beyond morphology prediction to application-specific outputs, including wet-state stability, release kinetics, retention of mechanical properties, antibacterial activity, and coating adhesion. However, their reliability will depend on sufficiently large and standardized datasets, consistent reporting of processing and environmental variables, model interpretability, and prospective experimental validation.

Validation should be defined according to the dominant failure mechanisms associated with the intended application. For wound-contact materials, relevant failure modes include excessive swelling or dissolution, loss of fibrous morphology, uncontrolled release, loss of wet-state mechanical integrity, inadequate control of microbial colonization, and insufficient support of tissue repair. In urological reconstruction, relevant constraints include dimensional instability, degradation under urine-relevant conditions, repeated deformation, insufficient compliance, reduced suture retention, and inadequate restoration of tissue-specific function. For implant coatings, coating-substrate delamination, cracking, premature degradation, loss of therapeutic function, biofilm formation, and insufficient corrosion protection represent relevant failure modes. These endpoints are not interchangeable, and favorable performance in a single category should not be considered sufficient evidence of overall suitability for the intended application. Material optimization should therefore determine whether improvement of one property compromises other functions that are critical to the intended biomedical interface.

The environmental performance of electrospun biomedical materials depends on feedstock production, solvent use, processing energy, and end-of-life management. Solvent substitution can reduce specific hazards, but its implications for fiber formation and material properties require formulation-specific assessment [130,131]. Energy demand also includes solution preparation, environmental control, drying, stabilization, and sterilization, and must be considered in relation to manufacturing throughput. Bio-derived feedstocks require consideration of extraction, purification, and chemical modification, while biodegradability must be assessed together with the environmental fate of degradation products and released additives. Biological contamination and limited component separability may restrict recycling of used biomedical materials; solvent recovery and manufacturing-waste reduction offer additional opportunities for resource conservation. Laboratory-scale LCA has demonstrated the dependence of electrospinning impacts on processing conditions [132]. For biomedical applications, environmental comparisons should additionally account for equivalent functional performance and service life, using explicitly defined functional units and system boundaries. Polymer origin, solvent selection and biodegradability alone therefore provide insufficient grounds for claiming overall sustainability.

**Figure 12:**
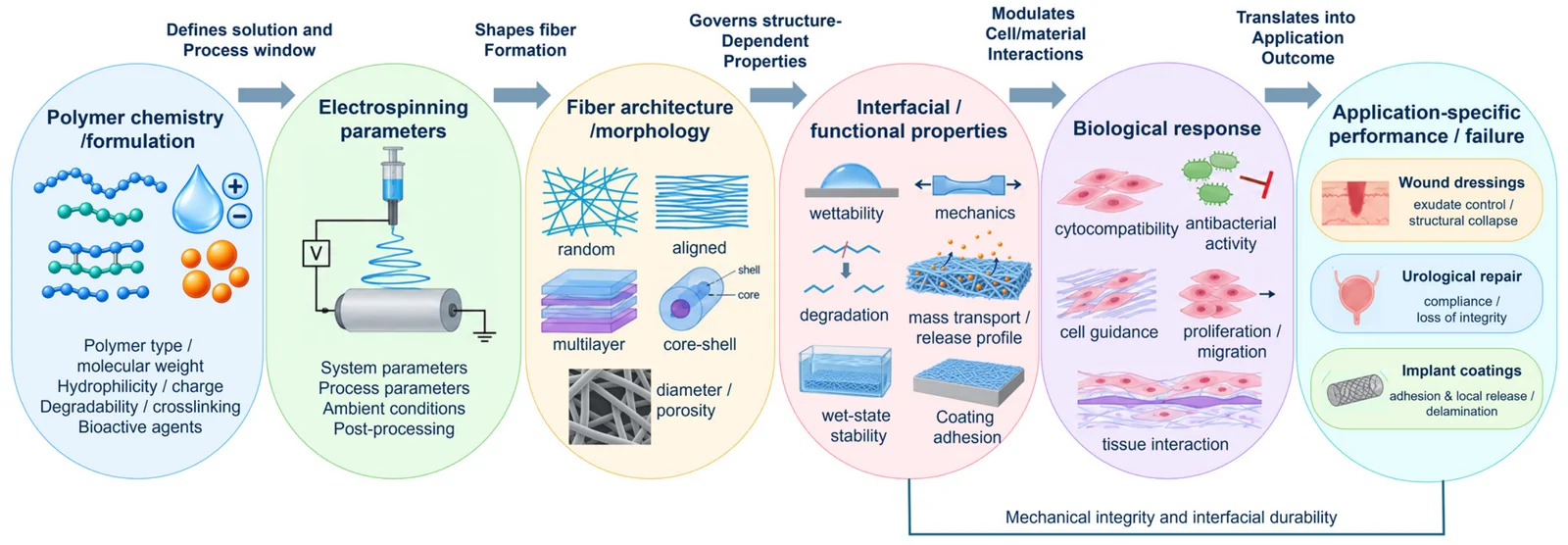
Relationships among material and processing inputs, fibrous architecture, interfacial properties and application-specific validation. Arrows indicate design relationships to be tested experimentally, rather than deterministic biological effects. Exposure and post-processing can modify architecture and interface properties. The three application examples identify distinct functions and failure modes; assay-specific and clinical evidence are not interchangeable.

Figure 12 integrates the design relationships considered in this review. Polymer chemistry and processing act jointly on architecture, while hydration, proteins, loading and post-processing modify the structure and interface encountered during use. Biological assays and application-specific tests provide separate evidence for compatibility, function and failure.

The most immediate research priority is to establish how hydration and exposure modify the functions required during use, by measuring structural evolution, mechanical retention and bioactive availability over matched observation periods. This assessment should be complemented by longitudinal studies linking material changes to tissue-specific outcomes, including barrier restoration, mechanical recovery and implant integration. Manufacturing studies must then determine whether the attributes responsible for these outcomes remain reproducible across batches and are preserved after sterilization and storage. Together, these priorities address the principal gap between demonstrating an initial material property and establishing a reproducible biomedical function.

## 7. Conclusions

Bioactive electrospun materials should not be evaluated solely according to fiber morphology, polymer composition, or the presence of incorporated functional components. Across the biomedical interfaces considered in this review, material performance depends on the extent to which fibrous architecture, mechanical behavior, interfacial stability, and bioactive function are retained under the chemical, mechanical, and biological conditions associated with the intended application. Wet-state behavior and application-specific failure modes should therefore be considered integral components of materials design rather than secondary validation endpoints.

The available evidence is not equally mature across the three application domains. Electrospun wound dressings are supported by a broad literature encompassing physicochemical characterization, functional in vitro studies, and multiple preclinical models, although direct comparison remains limited by substantial heterogeneity in formulations, testing protocols, and biological models. Urological applications remain less extensively validated, particularly with respect to prolonged urine exposure, cyclic deformation, barrier restoration, surgical handling, and tissue-specific function. Implant and biomedical-device coatings require a distinct validation framework in which biological or therapeutic performance must be considered together with coating-substrate adhesion, delamination resistance, wet-state durability, sterilization response, and, where relevant, corrosion and wear behavior.

The comparison identifies several recurring materials-design constraints. ECM-like fibrous morphology alone does not establish bioactivity. Functional claims should be supported by validation appropriate to the intended biological mechanism and application. Increased compositional or architectural complexity should be justified by a measurable and reproducible functional advantage. Process–structure–function relationships should be interpreted cautiously in multicomponent systems because formulation changes frequently affect several physicochemical variables simultaneously. Material optimization should also account explicitly for dominant failure mechanisms rather than focus exclusively on maximizing individual favorable properties.

The main challenge is therefore not the fabrication of increasingly complex electrospun biomedical materials, but the demonstration that their structural, mechanical, interfacial, and biological functions are retained to the extent and for the duration required by the intended application. Priority should be given to evaluating material stability and bioactive function under relevant physiological and mechanical conditions, and to relating changes in material properties to biological outcomes and application-specific failure modes. The effects of manufacturing, sterilization, and storage on material performance and batch-to-batch reproducibility also require further investigation. More consistent reporting of processing and testing conditions would facilitate comparisons across studies and support the assessment of electrospun materials for biomedical applications.

## Figures and Tables

**Table 1:** Process–structure–function relationships in electrospun bioactive nanofibers.

Design Dimension	Main Control Variables	Structural/ Physicochemical Outcome	Functional/ Biomedical Relevance	Critical Issue	Refs.
Polymer and solution properties	Molecular weight, concentration, viscosity, conductivity, surface tension, solvent system	Chain entanglement, jet stability, fiber diameter, defect formation, surface morphology and porosity	Control of fiber formation, wettability, interfacial properties, and payload distribution/release	Narrow processing window and high formulation sensitivity	[6,32,33,35,40,41,42,43,44,45]
Processing parameters	Applied voltage, flow rate, collecting distance	Jet stretching, solvent evaporation, fiber diameter, deposition uniformity and mat thickness	Reproducibility of morphology and material properties; consistency of subsequent mechanical and release behavior	Strong coupling among processing variables and limited transferability between formulations and setups	[32,33,34]
Spinneret and collector design	Single-fluid, coaxial or emulsion electrospinning; static, rotating, tubular or patterned collectors	Random, aligned, tubular and core–shell fiber architectures	Directional cell guidance, surface coverage, payload localization and release control	Architectural uniformity and reproducibility become increasingly difficult to maintain during scale-up	[13,33,46]
Ambient conditions	Temperature and relative humidity	Solvent evaporation, fiber solidification, phase separation, crystallization, surface morphology and porosity	Batch reproducibility and preservation of morphology and payload properties	Environmental sensitivity may produce significant batch-to-batch variability	[47,48,49]
Bioactive-agent incorporation	Type, concentration and localization of drugs, proteins, peptides, growth factors, nanoparticles or functional fillers	Changes in solution properties, fiber morphology, payload distribution and degradation behavior	Potential local therapeutic activity; controlled release; modulation of material–biological interactions	Burst release, payload deactivation, cytotoxicity and formulation instability	[27,45,54,55,56,57,58]
Post-processing, sterilization and manufacturing control	Crosslinking, thermal treatment, sterilization method, environmental control, scale-up conditions	Changes in morphology, crystallinity, molecular structure, mechanical behavior, degradation and payload stability	Preservation of critical material attributes and biological function before use	Post-processing, sterilization or scale-up may alter properties established during fabrication	[15,16,17]
Physiological exposure	Hydration, ionic strength, pH, proteins, enzymes and mechanical loading	Swelling, plasticization, degradation, surface rearrangement and loss of fibrous integrity	Retention of wet-state morphology, mechanical properties, release behavior and bioactivity	Dry-state characterization may not predict functional performance under use-relevant conditions	[14,15,16,17,27,54,55]

**Table 2:** Representative formulations, solvents, electrospinning and stabilization conditions, architectures and bioactive components.

Polymer Formulation	Solvent System	Electrospinning/ Stabilization	Fiber Architecture	Functional Component	Ref.
**A. Wound dressings and skin regeneration**					
PCL/gelatin, 7:3	HFIP + formic acid	15 kV; 0.7 mL/h; 14 cm	Blend fibers	Arbutin, nominal 5 or 10 wt%	[60]
PLGA 50:50, 20% *w*/*v*; ZnO 1% *w*/*v*	HFIP; aqueous VEGF core	10–12 kV; shell 1/core 0.1 mL/h; 12–14 cm; 25 °C, 50% RH	Coaxial core–shell	ZnO in shell; VEGF in core	[18]
PCL 12% *w*/*v*; collagen I coating 0.1% *w/v*	DCM/DMF, 7:3	17 kV; 0.6 mL/h; 28 cm; air plasma 6.8 W, 5 min before collagen	Surface-functionalized fibers	Collagen I; Ag nanoparticles	[65]
SF/collagen, 85:15, 5% *w*/*v*; PVA/chitosan, 80:20	HFIP; aqueous acetic acid for PVA/chitosan	14 kV (SF/collagen), 8 kV (PVA/chitosan); each 1 mL/h; 15 cm; GTA vapor 12 h	Co-electrospun networks	Vitamin D_3_; CeO_2_ nanoparticles	[70]
Gelatin 35% + chitosan 2% (*w*/*v*), 70:30; PCL 14% *w*/*v*	Acetic acid/water, 50:50; chloroform/DMF, 3:1	19 kV, 0.3 mL/h (gelatin/chitosan); 25 kV, 1 mL/h (PCL); 10 cm; 25 ± 1 °C, 45% RH	Co-electrospun fibers	Ag-coated CQDs; citrate in GCP–QC	[73]
PVA 7 wt%; HA 0.25 wt%; CA 0.25 wt%	Water	20 kV; 0.5 mL/h; 10 cm; 150 °C, 2 h	Crosslinked blend fibers	HA	[14]
Eudragit L100/PVP, 70:30; total 12% *w*/*v*	Ethanol/water, 80:20	16 kV; 1 mL/h; 10 cm; 25 °C, 30% RH	Blend fibers	Propolis 5, 10 or 20 wt%	[78]
PLCL 8% *w*/*v*/nZnO sheath; CEFFE/5% HA core, 7:3 *v*/*v*	HFIP sheath; aqueous HA-containing core	20 kV; shell 1/core 0.2 mL/h; 15 cm; 27 °C, 60% RH; vacuum dry 72 h	Coaxial core–shell	nZnO, 2 wt%; CEFFE; HA	[76]
Sheath: gelatin 20% *w*/*v*; core: PVA 12% + gelatin 10% solutions, 9:1 *v*/*v*	Sheath: water/DMF/formic acid, 50:25:25; core: water	17–19 kV; feeds 1 mL/h; 12 cm; 30% RH; 25% aqueous GTA vapor 24 h	Coaxial core–sheath	Phenytoin 20 mg/mL in sheath; lysozyme 5 mg/mL in core	[79]
**B. Urological repair and regeneration**					
ELP/GelMA: 5% *w*/*v* each (5E5G)	HFIP	25 kV; 1 mL/h; 13 cm; 1% Irgacure 2959/ethanol, 2 h; UV 6.9 mW/cm^2^, 600 s	Photocrosslinked blend fibers	Protein-derived matrix; no exogenous drug	[80]
SF 33 wt%; combined with 3D-BAMG	Water	20 kV; 1.2 mL/h; 10 cm; drum 2000 rpm; 25 ± 1 °C, 50 ± 5% RH; stabilize 37 °C, 90% RH, 36 h	Aligned microfibers in decellularized graft	SDF-1α	[81]
PLGA/collagen I, 7:3; total 15% *w*/*v*; MeHA hydrogel	HFIP for fibrous layer	25 kV; 1 mL/h; 15 cm; 19G; vacuum dry 24 h; MeHA photocrosslinking	Fiber/hydrogel bilayer	Urethral epithelial and smooth-muscle cells	[82]
PCL/gelatin, 7:3; total 10% *w*/*v*	HFIP + 0.2% *v*/*v* acetic acid	15 kV; 1.5 mL/h; 10 cm; mandrel 60 rpm; vacuum dry ≥ 1 week	Composite fibers	MgO, 0.1/0.5/1.0% *w*/*v*; selected PGM-0.5	[83]
**C. Implant and biomedical device coatings**					
PLA 10% *w*/*v*; nHAp 5–10% *w*/*v* on Ti	Chloroform/acetone, 1:1	15 kV; 2 mL/h; 10 cm	Direct-deposited composite fibers	nHAp	[25]
PCL, 20%; BMP-2-loaded CNPs; α-tocopherol	DCM/methanol, 3:1	10 kV; 5 mL/h; 11 cm; 21G; 22 °C, 48–50% RH	Nanoparticle-containing fibers on Ti	BMP-2; α-tocopherol	[77]
PCL and PLGA 50:50, each 20% *w*/*v*; antibiotic solutions added	TFE; Rif in chloroform; Van in DMSO	16 kV; 0.5 mL/h; 8 cm; coaxial shell:core 2:1; vacuum dry 1 week	Bilayer/coaxial fibers within Ti lattice	Vancomycin/PCL inner; rifampicin/PLGA outer	[84]
PCL 5%; gelatin 0.5%; DEX 0.032%; β-GP 0.5%; AA and HAp each 0.04% (*w*/*v*)	2,2,2-Trifluoroethanol	15 kV; 0.34 mL/h; 13 cm; Ti screw on rotating shaft	Composite fibers directly on Ti screw	HAp; dexamethasone; β-glycerophosphate; ascorbic acid	[85]
PCL and gelatin 10% *w*/*v* solutions, 4:1; selected EGCG 0.5% *w*/*v*, Rif 0.05% *w*/*v*	2,2,2-Trifluoroethanol	17 kV; 1 mL/h; 15 cm; 3 h; Tris-HCl pH 8.5, 6 h; vacuum dry 24 h	EGCG-containing composite fibers on Ti	EGCG; rifampicin	[86]

HFIP, hexafluoroisopropanol; DCM, dichloromethane; DMF, N,N-dimethylformamide; GTA, glutaraldehyde; ELP, elastin-like polypeptide; GelMA, gelatin methacryloyl; SF, silk fibroin; CA, citric acid.

**Table 3:** Application-specific design requirements and validation endpoints for bioactive electrospun fibers.

Application	Biological/ Interface Environment	Primary Material Requirements	Representative Electrospun Strategies	Critical Validation Endpoints	Refs.
**Wound dressings and skin regeneration**	Exudate, handling and potential infection	Fluid management; conformability; hydrated integrity; controlled delivery	Stabilized hydrophilic mats; core–shell delivery; responsive functional phases	Fluid uptake and vapor transport; wet morphology/mechanics; release and retained activity; microbial assays; wound repair	[14,19,76,100,101]
**Urological** **repair and** **regeneration**	Bladder/urethra: hydration, urine and deformation. Cavernous nerve repair: a distinct perineural interface	Bladder/urethra: compliance, barrier function and surgical integrity. Nerve repair: neural functional recovery	Fiber–hydrogel multilayers or bilayers; suturable protein-derived mats; endogenous NGF recruitment	Urine exposure, cyclic mechanics, suture retention and barrier restoration for bladder/urethra; neural and erectile-function endpoints for cavernous nerve repair	[21,22,80,82,102,103]
**Implant and biomedical device** **coatings**	Solid substrate–tissue interface; insertion/loading; microbial and, where relevant, electrochemical exposure	Coating adhesion; interfacial persistence; tissue integration; controlled therapeutic function	Polymer/ceramic composite coatings; osteogenic payloads; antibiotic-containing multilayers	Insertion retention, adhesion/delamination and wear; hydrated stability; release; biofilm assays; tissue integration; corrosion where relevant	[23,24,25,61,62,77,84,104,105]

**Table 4:** Selected physicochemical and biological outcomes of electrospun materials with testing conditions and evidence models.

Material/ Formulation	Fiber Morphology	Mechanical/ Stability Endpoint	Bioactive Component/Release	Biological Model/ Evidence	Ref.
**A. Wound dressings and wound-related mechanisms**					
PVA/HA/CA; 150 °C, 2 h. UV-C/cell assays	Random fibers; as-spun diameter 130.2 ± 50.3 nm; SEM-image porosity 54.37%	Dry E: 72.73 ± 7.45 → 237.96 ± 14.66 MPa after crosslinking. Mass loss: 2.96 ± 0.45% (pH 7.4, 37 °C, 48 h). WVTR after UV-C: 2492.6 ± 21.6 g m^−2^ day^−1^ (37 °C; RH < 40%; PBS pH 8)	HA 0.25 wt% in formulation; no agent-release endpoint included	24 h in vitro (s.e.m.): HEK293 PI^+^ 7.17 ± 0.17% (control), 5.18 ± 1.25% (plastic-adherent), 7.40 ± 1.35% (suspended); ns. HaCaT PI/PKH67 intensity ratio: 8.54 ± 1.43 vs. 7.56 ± 0.67; ns. Limited stable adhesion	[14,95]
GCP; GCP–Q (Ag-CQDs); GCP–QC (Ag-CQDs/citrate)	Co-electrospun fibers; diameter 25–300 nm. Liquid-displacement porosity: 85%, 87%, 88%, respectively	Water contact angle: 107°, 67°, 53°, respectively. Modified mats lost mass faster in PBS (pH 5 and 7.5)	Ag-CQDs and citrate; no quantitative loading or release value included	Animal: rat full-thickness wounds, days 4, 8 and 12; histological re-epithelialization with GCP–Q/GCP–QC	[73]
Eudragit L100/PVP (70:30); nominal propolis 5, 10 or 20 wt%	Blend fibers; unloaded diameter 326 nm; density-based porosity ≈ 75%. PRO10 diameter 353 nm	UTS: 9.1 ± 2.7 MPa unloaded → 10.1 ± 0.45 MPa PRO20; elongation 44.2 ± 3.2% → 20.4 ± 0.6%. 0.04 mm/s; *n* = 3; hydration unspecified	PBS pH 7.4, 37 °C; 4 cm^2^ mat; 20 h release ≈28%, 37%, 55% for PRO5/10/20, respectively; UV 325 nm	24 h disk diffusion: PRO20 inhibition zones 13.5 mm (*E. coli*), 15.3 mm (*S. aureus*). NIH3T3 MTT/live–dead/adhesion, days 1/3/5; no animal wound model	[78]
PLCL/nZnO sheath; HA/CEFFE core; 2 wt% nZnO	Coaxial core–shell fibers	Dry UTS: 3.70 ± 1.20 MPa vs. 5.10 ± 0.40 MPa without nZnO (20 °C; 65% RH; 10 mm/min; *n* = 5)	Zn^2+^ and bFGF/TGF-β1/VEGF release up to 96 h. Growth factors: 1 cm^2^ in 2 mL PBS, 37 °C, replacement sampling; ELISA, *n* = 3. Numerical Zn^2+^ amount omitted because source units are inconsistent	In vitro: OD_600_ growth reduction ≈40% *E. coli* and ≈50% *S. aureus* at 24 h; NIH3T3 metabolic activity and scratch closure	[76]
GTA-crosslinked gelatin/phenytoin sheath; PVA/gelatin/lysozyme core	Coaxial fibers; SEM diameter 213 ± 80 nm. Most fibers collapsed by day 14 in PBS	Dry → PBS-immersed: E 173 ± 3 → 5.95 ± 0.70 MPa; UTS 4.98 ± 2.00 → 1.80 ± 0.10 MPa. 1 mm/min; *n* = 3; pre-soak duration unspecified. Mass loss 52.9 ± 5.6%, PBS 37 °C, 28 d	10 mg/3 mL PBS pH 7.4, 37 °C, 150 rpm, replacement sampling. Lysozyme: 83.5% at 7 d vs. ≈90% at 8 h from core-only fibers	L929 MTT ≈ 90 ± 2%; days 1/3/5. In vitro compatibility and release; no animal wound-repair endpoint	[79]
**B. Urological repair and regeneration**					
PLGA/collagen I (7:3) membrane + cell-seeded MeHA hydrogel	Membrane/hydrogel bilayer; membrane diameter 144 ± 38 nm; porosity 90.99 ± 3.39%	Integrity lost at ≈52 d in artificial urine, 37 °C. Reported tensile stress > 0.4 MPa; conditioning unspecified	Rabbit urethral epithelial and smooth-muscle cells in MeHA	Rabbit urethral-defect reconstruction; urethrography and histology at 3 months; cell-seeded and comparison groups	[82]
Photocrosslinked ELP/GelMA (5E5G) vs. GelMA (0E10G)	Fibrous mat; diameter 790 ± 40 vs. 1260 ± 50 nm; porosity 18.91 ± 1.35% vs. 21.00 ± 0.17%	5E5G: E 25.63 ± 4.77 kPa; UTS 41.00 ± 6.68 kPa; elongation 167.4 ± 5.41%. DPBS-hydrated suture retention 13.29 ± 0.75 vs. 7.41 ± 0.97 kPa (s.e.m., *p* < 0.01). >50% mass loss, DPBS 37 °C, 21 d	Protein-derived matrix; no exogenous therapeutic payload	Human LUT cells in vitro; rat subcutaneous host response to day 56, rather than LUT reconstruction	[80]
PCL/gelatin, 7:3; MgO 0.5% *w*/*v* in spinning solution (PGM-0.5)	MgO-containing composite fibers	UTS > 50% higher than filler-free control after 1 h in physiological saline; 10 mm/min, *n* = 3	Mg^2+^ release followed for 14 d in 0.9% saline, 37 °C; replacement sampling, ICP-OES, *n* = 3	CFU reductions >90% *E. coli*, >85% *S. aureus* (24 h). Urothelial/smooth-muscle CCK-8 ≈40% higher at day 7. Rat 10 mm bladder defect: cystography/histology at 12 wk; mean ± SEM	[83]
**C. Implant and biomedical-device coatings**					
PLA 10% *w*/*v* + nHAp 10% *w*/*v* on Ti; neat PLA comparator	Random fibers; diameter 800 ± 200 vs. 760 ± 200 nm; SEM-image porosity 50% vs. 51%	EIS-fitted Rcorr: (7.42 ± 0.18) × 10^6^ vs. (2.65 ± 0.25) × 10^5^ Ω. PBS, room temperature; 1 h equilibration; 10 mV; 0.01 Hz–100 kHz; *n* = 5	nHAp mineral phase	Electrochemical barrier assessment; biological integration is not an endpoint of this comparison	[25]
BMP-2-loaded CNPs + α-tocopherol in PCL on Ti	Nanoparticle-containing electrospun coating	—	CNP encapsulation 76.5 ± 1.01%; BMP-2 on implant 94 ± 2.98 ng at 60 s deposition. BMP-2: ≈70%/35 d in fibers vs. 99%/5 d in CNPs; PBS +0.05% Tween 20 +0.1% BSA. α-Tocopherol: ≈60%/24 h; PBS +2% Tween 20. 37 °C, 100 rpm	In vitro: WST-1 >85% of control (L929/MC3T3-E1, 24/72 h); MC3T3-E1 ALP up to 16-fold higher (7/14 days)	[77]
Ti lattice with PCL/vancomycin–PLGA/rifampicin fibers	Bilayer and coaxial architectures	Ex vivo: coating retention during press-fit insertion	500 μL PBS pH 7.4, 37 °C, refreshed at sampling. Bilayer, day 42: Rif 62.52 ± 1.79%; Van 79.59 ± 3.30%; UPLC	*S. aureus* planktonic/adherent CFU after pre-elution to day 42; 24 h challenge, *n* = 3. MC3T3-E1 assays to 14 d. Cadaveric rat tibial insertion; no living-animal infection model	[84]
PCL/gelatin/HAp plus dexamethasone, β-glycerophosphate and ascorbic acid on Ti	Random composite coating; SEM diameter 187 ± 23 nm	—	Multicomponent osteogenic formulation; the response cannot be assigned to HAp alone	Animal: rabbit bone–implant contact at 12 weeks, 50.90 ± 9.75% coated vs. 45.43 ± 3.67% bare Ti	[85]
EGCG-modified PCL/gelatin + rifampicin on Ti	Surface-modified electrospun coating	Peel: 583.43 ± 38.19 N/m (0.5% EGCG + alkaline treatment) vs. 66.7 ± 13.1 N/m (unmodified, before treatment); 10 mm/min, n = 4. Wear 27 ± 4 cycles: 600-grit SiC, 50 g load	Rif 38.28 ± 2.88% at 3 d; 68.46 ± 3.29% at 28 d. 4 cm^2^/4 mL PBS pH 7.4, 37 °C, 100 rpm; HPLC, replacement sampling	MC3T3-E1 cytocompatibility/osteogenic assays; antibacterial assays. Rat *S. aureus* subcutaneous implant infection: early CFU and day-7 histology; not dental loading	[86]

CA, citric acid; CEFFE, cell-free fat extract; CNPs, chitosan nanoparticles; CQDs, carbon quantum dots; DPBS, Dulbecco’s phosphate-buffered saline; E, tensile elastic modulus; EGCG, epigallocatechin gallate; ELP, elastin-like polypeptide; EIS, electrochemical impedance spectroscopy; GelMA, gelatin methacryloyl; HA, hyaluronic acid; HAp, hydroxyapatite; MeHA, methacrylated hyaluronic acid; nZnO, zinc oxide nanoparticles; PBS, phosphate-buffered saline; PCL, polycaprolactone; PI, propidium iodide; PLCL, poly(L-lactide-co-ε-caprolactone); PLGA, poly(lactic-co-glycolic acid); PVP, polyvinylpyrrolidone; PRO, propolis; PVA, poly(vinyl alcohol); Rif, rifampicin; SEM, scanning electron microscopy; s.e.m., standard error of the mean; ns, not statistically significant; RH, relative humidity; LUT, lower urinary tract; GCP, gelatin/chitosan/PCL; Rcorr, fitted corrosion resistance; UTS, ultimate tensile strength; WVTR, water vapor transmission rate.

## Data Availability

No new experimental data were generated in this study. The quantitative data summarized in the comparative tables were obtained from the cited publications. Data sharing is not applicable to this article.
